# Stochastic journeys of cell progenies through compartments and the role of self-renewal, symmetric and asymmetric division

**DOI:** 10.1038/s41598-024-63500-2

**Published:** 2024-07-15

**Authors:** Hanan Dreiwi, Flavia Feliciangeli, Mario Castro, Grant Lythe, Carmen Molina-París, Martín López-García

**Affiliations:** 1 School of Mathematics, University of Leeds, Leeds, UK; 2 Systems Pharmacology and Medicine, Bayer AG, Leverkusen, Germany; 3 Grupo Interdisciplinar de Sistemas Complejos (GISC), Instituto de Investigación Tecnológica (IIT), Universidad Pontificia Comillas, Madrid, Spain; 4 Los Alamos National Laboratory, Theoretical Biology and Biophysics Group, Theoretical Division, Los Alamos, NM USA

**Keywords:** Applied mathematics, Computational biology and bioinformatics

## Abstract

Division and differentiation events by which cell populations with specific functions are generated often take place as part of a developmental programme, which can be represented by a sequence of compartments. A compartment is the set of cells with common characteristics; sharing, for instance, a spatial location or a phenotype. Differentiation events are transitions from one compartment to the next. Cells may also die or divide. We consider three different types of division events: (i) where both daughter cells inherit the mother’s phenotype (self-renewal), (ii) where only one of the daughters changes phenotype (asymmetric division), and (iii) where both daughters change phenotype (symmetric division). The self-renewal probability in each compartment determines whether the progeny of a single cell, moving through the sequence of compartments, is finite or grows without bound. We analyse the progeny stochastic dynamics with probability generating functions. In the case of self-renewal, by following one of the daughters after any division event, we may construct lifelines containing only one cell at any time. We analyse the number of divisions along such lines, and the compartment where lines terminate with a death event. Analysis and numerical simulations are applied to a five-compartment model of the gradual differentiation of hematopoietic stem cells and to a model of thymocyte development: from pre-double positive to single positive (SP) cells with a bifurcation to either SP4 or SP8 in the last compartment of the sequence.

## Introduction

Humans, animals, plants, and even fungi consist of multiple cell types which maintain the organism in homeostasis. All cells share the same DNA but gene expression variation lead to a range of different cell functions. Cell differentiation, controlled through changes in gene expression, enables cells to become more specialised. In embryonic development, non-specialised cells give rise to differentiated and functional cells^1,2^. Sequences of progressive cell-type (or phenotype) changes also take place in adult organisms, leading to cell populations with specific functions. Populations of “stem cells” retain the potential to develop into many different types. Generally speaking, we can say that “stem-like” cells are able to differentiate into multiple cells of a lineage, giving rise to more mature cells. Mathematical models of cell differentiation, fitted to experimental data, have been used to estimate per-cell event rates, to study differentiation timelines, and cellular dysregulation: keratinocyte differentiation and psoriasis pathogenesis^3–5^, human gastrointestinal tract cellular differentiation^6,7^, blood-cell differentiation and hematopoiesis^8–11^, cancer differentiation as in chronic myeloid leukaemia^12^, T-cell development and thymic selection^13–15^, and the T cell exhaustion process^16–20^.

Differentiation involves a series of cell phenotypes, and may involve different spatial locations as well, as in keratinocyte differentiation; the epidermis, the outermost skin layer, is a stratified collection of cells, called keratinocytes, characterised by several differentiated stages. During its lifespan, a keratinocyte transits from a more internal to an external stratum, undergoing biochemical and morphological changes^21^. A similar process is that of colonic cell differentiation; in this case, the endothelial tissue of the human gastrointestinal tract consists of several cell types with different functions, from an outermost layer that acts as a protective barrier and absorbs nutrients, to an inner one of proliferative stem-like cells. In Ref.^6^, the authors investigate cell proliferation and motion in the intestinal crypt (invaginations typical of the colonic tract). Differentiation hierarchies are also found in healthy and cancer systems, where a tumour cell population may arise from cellular mutations. In Ref.^12^, a mathematical model of the haematopoietic system representing stem-like cells, progenitors, differentiated and terminally differentiated cells is considered. This hierarchy describes both normal and leukaemic cells (cancer cells) with differences in cellular rates; for instance, leukaemic stem-like cells rapidly divide by self-renewal compared to other differentiated cell types. The authors in Ref.^22^ study the plasticity of cancer cells with high and low tumorigenesis potential, making use of a model where cancer cells undergo division to replenish high potential cells or generate cancer cells with lower tumorigenic potential. Cellular de-differentiation also needs to be considered. In chronic infection or cancer, CD8^+^ T cells (or immune cytotoxic cells) have been observed to acquire dysfunctional (or exhausted) properties^23^. Recent studies have brought a greater understanding of this exhaustion process^20^. Yet, we still dot not completely understand the molecular and epigenetic mechanisms that lead to T cell exhaustion. Unanswered questions include when and at which stage exhaustion becomes irreversible, and to what extent exhausted cells can be re-invigorated.

Advances in genetic labelling^24^ have uncovered different types of cell division, which impact the ability of a cell pool to expand or contract. Fate mapping studies reveal linear model structures of cell differentiation, such as the hematopoieitic system^9^. Different division events drive a range of biological processes according to whether a given cell population grows by one new cell (self-renewal), stays the same size (asymmetric division, where one of the daughter cells changes phenotype), or shrinks (symmetric division, where both daughter cells change phenotype)^9^. In such hierarchical systems cells might not be characterised by the same rates throughout the differentiation process^9,25^; an example is found in the hematopoietic system, where a turning point has been observed before the multi-potent progenitor (MPP) stage, where self-renewal is reduced and cell differentiation increased^9^. The heterogeneity of stem-cell populations and their progeny might derive from environmental and intra-cellular perturbations that are still poorly understood. For example, it is unknown whether hematopoietic stem cells (HSCs) undergo symmetric or asymmetric cell division in the unperturbed bone marrow^8–10^. The authors of Ref.^8^ found fundamental differences between the normal maintenance of the haematopoietic system, its regulation by challenge, and its re-establishment after transplantation, suggesting that different per-cell rates (of differentiation, death, or division) apply to a given scenario.

Recent experimental techniques have made it possible to track individual cell states (or compartments) and the progeny of a single cell^26,27^. Mathematical and computational approaches have increased our quantitative understanding of cell population dynamics. Deterministic models, which do not incorporate randomness and are typically easier to analyse, may describe the mean dynamics of such populations. However, single-cell behaviour is invariably stochastic. Deterministic compartmental models are used in ecology and cell biology, and in pharmaco-kinetics/pharmaco-dynamics, where they describe the concentration-versus-time curves of a drug following administration and how the drug can influence reaction rates and fluxes. Compartments can represent populations across different scales, from the intra-cellular to the whole-organ level. A single compartment can be thought of as a collection of items, agents, or individuals acting in a homogeneous fashion; agents can differ by form or location, so that a compartment might represent the concentration of a drug in blood or in a given organ.

A multi-compartmental model consists of two or more interconnected compartments and it encodes changes of state (e.g., precursor to product cells) or changes of location. Multi-compartmental models, where compartments are arranged in a sequence or hierarchy, have been used to model cell division and differentiation, from deterministic^28–34^ and stochastic^35–38^ perspectives. Many of these models have been developed in the context of cancer^30–32,34^, and have considered specific hierarchies or parameter ranges (e.g., tumour dynamics with no asymmetric division^30^, colorectal crypt^31^, multiple mutations^29^, age-structured models^34^, or Moran-type dynamics^32^). Stochastic models are less frequent. Dingli et al.^36^ proposed a mathematical model to illustrate the role of mutations on stem-cell division and the development of tumours. Shahriyari and Komarova^38^ proposed a stochastic model for a renewing tissue, addressing the optimisation problem of tissue architecture in the context of mutant production. Clayton *et al.*^35^ discussed the classical epidermal proliferation unit (EPU) model of adult epidermal homeostasis, and proposed a single proliferative compartment to better explain experimental observations. Mamis *et al.*^37^ considered a three-type branching process to model the dynamics of cell populations in colonic crypts. Refs.^33,39^ are recent reviews in this field. The references above differ from the multi-compartmental model analysed here, since we explicitly consider both asymmetric division and cellular de-differentiation. This is not only a generalisation of previous efforts, but it enables us to examine several differentiation processes, such as CD8^+^ T cell exhaustion reversibility and cancer cell tumorigenesis. Our approach allows the parameters of each cell population (or compartment) to differ, and we do not restrict ourselves to a uni-directional cell flow toward more differentiated states. Thus, we are able to investigate properties caused by the heterogeneity of the different cell populations (or compartments) in a linear-structure model of stem cell differentiation. We also go beyond the more frequent deterministic approach, and analyse the stochastic dynamics of the population with probability generating functions. Finally, by analysing the stochastic dynamics at the single-cell level, we obtain predictions which could be tested (or used for parameter calibration) with novel single-cell experimental methods.

In some circumstances the mean dynamics of cell differentiation might be captured by ordinary differential equations; however, details about cell division, death or differentiation are often better modelled by a stochastic processes, capable of capturing aspects at the single-cell level, genetic and intra-cellular processes, or describing scenarios where a cell population is descended from a few progenitor cells. Within the field of stochastic analysis, the theory of branching processes has been widely used in cell dynamics^40^. For instance, the classic Galton-Watson model, which was originally developed to study the extinction of family surnames^41^, has been successfully applied to quantify the progeny of a cell^40^. The theory of branching processes can answer questions related to the limiting behaviour (e.g., probability of extinction or unbounded growth) of cell populations. A natural generalisation is the multi-type branching process, where cells are not all of the same type. These models can effectively represent cells changing their spatial location^42^, or their phenotype^43^ over time. They characterise cell dynamics across compartments, where cells in the same compartment follow identical rules. We highlight the seminal work by Matis^44^, who proposed a stochastic compartmental model of cell dynamics.

We put forward a general mathematical model with a sequence of *N* compartments, characterising the process by which, from a stem-cell pool (in compartment $C_1$), cells undergo differentiation events across adjacent compartments, producing a terminally differentiated progeny population (in compartment $C_N$). Cells in each compartment can divide, die or transit to adjacent compartments (e.g., representing potentially reversible differentiation or phenotype change). In our stochastic model, differentiation can either be reversible (e.g., cancer cell mutations) or irreversible (e.g., embryonic cell development). Since cells in many tissues are short-lived compared to the life span of the host, a continuous regeneration process is required to maintain cell populations. We consider a close catenary system where all cell populations are explicitly modelled. Any cell generated in the sequence of compartments eventually dies or reaches the final compartment of product cells. However, as we show via a case study in Section “Tracking a thymocyte during its development”, alternative compartmental structures (for other potential biological applications) could be considered, for which many of our techniques (e.g., the analysis in Section “Lifeline analysis”) can be easily generalised. For example, a cyclic system can be defined if the last compartment is connected to the first one, or a so-called mammillary system, used to represent the blood compartment connected to each organ in the body or cellular states^42^.

The paper is organised as follows. In Section “Stochastic compartmental model” we describe the continuous-time Markov dynamics of cells dividing, dying or exiting across a sequence of compartments. The mean behaviour of the system is analysed in Section “Mean number of cells in each compartment”. In Section “The progeny of a single progenitor cell”, we study the proliferative potential of the system by computing the number of cells in the progeny of a single progenitor cell. In both sections we consider either the situation where differentiation events can be reversible, or a mathematically more simple “irreversible model”, where differentiation to the next compartment cannot be reversed. In Section “Lifeline analysis” our focus is a number of summary statistics related to a cellular lifeline which we track over time. In Section “Results”, we summarise numerical results inspired by biological applications to illustrate our approach and methods, and examine the impact of asymmetric and symmetric division on the cell population arising from a single progenitor.

## Stochastic compartmental model

We propose a stochastic model of cell division, death and differentiation across an ordered sequence of compartments. Cells in a given compartment may represent a common spatial location within the body, or a common phenotype (i.e., representing a biological state, defined by morphology and/or function). In practice, this means that cells in a compartment behave equally, with the same per-cell rates for a given division, death, or differentiation event. We consider a sequence of compartments $C_i$, $i\in \{1,\ldots ,N\}$, which cells follow, behaving independently of each other, as generally assumed in the theory of continuous-time branching processes^40^.

Our general stochastic model considers a number of cellular events inspired by some recent mathematical models^3,9,14,42^. We assume that each of these events takes place at a given per-cell rate. Particular situations of interest arise from setting some of the rates equal to zero, so that those events cannot take place, as we illustrate for three different case studies in Section “Results”. Each cell in a given compartment, $C_i$, can divide, die or exit to one of the two adjacent compartments. When a division occurs, both daughter cells might belong to the same compartment as the mother (this event is referred to as *self-renewal*), both daughter cells might instantaneously move to the next compartment (*symmetric division*), or one daughter cell might belong to the same compartment as the mother, and the other to the next compartment (*asymmetric division*)^3,9^.

**Figure 1:**
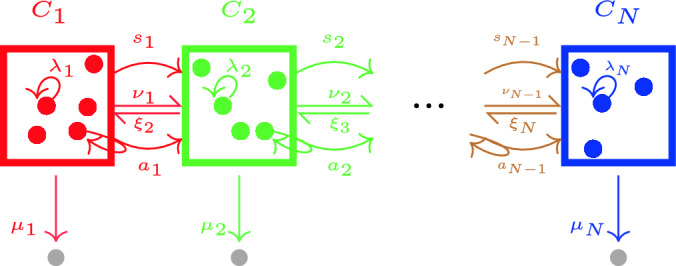
General stochastic model of cell division, death and differentiation for an ordered sequence of compartments. Grey cells represent a death event. Self-renewal events take place with rate $\lambda_i$, symmetric division events with rate $s_i$, and asymmetric division events with rate $a_i$. Differentiation events happen with per-cell rate $\nu_i$ (forward) or $\xi_i$ (backward). Death events have per-cell rate $\mu_i$. Each per-cell rate is a real positive number.

The stochastic model, shown in Fig. 1, is a continuous-time Markov chain (CTMC) ${{{\mathcal {X}}}}=\{({\varvec{C}}_1(t),{\varvec{C}}_2(t),\ldots ,{\varvec{C}}_N(t)):\ t\ge 0\}$, where ${\varvec{C}}_i(t)$ is a random variable that represents the number of cells in compartment $C_i$ at time *t*, with state space given by $\mathcal{S}=\{0,1,2,\ldots \}^N={\mathbb {N}}_0^N$. At any given time, the process can be at a particular state $(n_1,\ldots ,n_N)\in {{{\mathcal {S}}}}$, so that there are $n_i$ cells in compartment $C_i$. Thus, cell events, labelled E1 to E5 below, represent transitions between states as follows:

1. Self-renewal (cell division where both daughter cells remain in the same compartment as the mother) can occur in any compartment $C_i$, with per-cell rate $\lambda _i$, for $i\in \{1,\ldots ,N\}$,
  $$\begin{aligned} (n_1,\ldots ,n_{i-1},n_i,n_{i+1},\ldots ,n_N)\rightarrow_{\lambda_i n_i}\rightarrow & ~ (n_1,\ldots ,n_{i-1},n_i+1,n_{i+1},\ldots ,n_N) \nonumber . \end{aligned}$$
  The previous notation indicates that the event moving the process from state $(n_1,\ldots ,n_{i-1},n_i,n_{i+1},\ldots ,n_N)$ to state $(n_1,\ldots ,n_{i-1},n_i+1,n_{i+1},\ldots ,n_N)$ happens at rate $\lambda _i n_i$, and represents self-renewal.
2. Symmetric division (cell division where both daughter cells instantaneously move to the next compartment) can occur in compartment $C_i$ with per-cell rate $s_i$, for $i\in \{1,\ldots ,N-1\}$,
  $$(n_{1} , \ldots ,n_{{i - 1}} ,n_{i} ,n_{{i + 1}} , \ldots ,n_{N} ) \rightarrow_{s_i n_i}\rightarrow ~ (n_{1} , \ldots ,n_{{i - 1}} ,n_{i} - 1,n_{{i + 1}} + 2, \ldots ,n_{N} ).$$
3. Asymmetric division (cell division where one of the daughter cells remains in the same compartment as the mother, while the other goes to the next compartment) can occur in compartment $C_i$ with per-cell rate $a_i$, for $i\in \{1,\ldots ,N-1\}$,
  $$\begin{aligned} (n_1,\ldots ,n_{i-1},n_i,n_{i+1},\ldots ,n_N) \rightarrow_{a_i n_i}\rightarrow & ~ (n_1,\ldots ,n_{i-1},n_i,n_{i+1}+1,\ldots ,n_N). \end{aligned}$$
4. Differentiation (or migration) between adjacent compartments can occur with per-cell rate $\nu _i$, for $i\in \{1,\ldots ,N-1\}$ and $\xi _i$, for $i\in \{2,\ldots ,N\}$,
  $$\begin{aligned} (n_1,\ldots ,n_{i-1},n_i,n_{i+1},\ldots ,n_N) \rightarrow_{\nu_i n_i}\rightarrow & ~ (n_1,\ldots ,n_{i-1},n_i-1,n_{i+1}+1,\ldots ,n_N),\\ (n_1,\ldots ,n_{i-1},n_i,n_{i+1},\ldots ,n_N) \rightarrow_{\xi_i n_i}\rightarrow & ~ (n_1,\ldots ,n_{i-1}+1,n_i-1,n_{i+1},\ldots ,n_N). \end{aligned}$$
5. Cells can die in any compartment $C_i$ with per-cell rate $\mu _i$, $i\in \{1,\ldots ,N\}$,
  $$\begin{aligned} (n_1,\ldots ,n_{i-1},n_i,n_{i+1},\ldots ,n_N) \rightarrow_{\mu_i n_i}\rightarrow & ~ (n_1,\ldots ,n_{i-1},n_i-1,n_{i+1},\ldots ,n_N). \end{aligned}$$

We assume that cells in the last compartment, $C_N$, cannot symmetrically or asymmetrically divide, or differentiate to the next compartment. All rates are assumed to be positive real numbers.

### Mean number of cells in each compartment

We first study the dynamics of the mean number of cells in each compartment at time *t*, ${\mathbb {E}}[{\varvec{C}}_{i}(t)]$, described by the following system of ordinary differential equations^44^

1 $$\begin{aligned} \frac{d\, {\mathbb {E}}[{\varvec{C}}_{1}(t)]}{dt}= & {} -(\mu _1+\nu _1+s_1-\lambda _1) {\mathbb {E}}[{\varvec{C}}_{1}(t)] + \xi _2{\mathbb {E}}[{\varvec{C}}_{2}(t)], \nonumber \\ \frac{d\, {\mathbb {E}}[{\varvec{C}}_{i}(t)]}{dt}= & {} (\nu _{i-1}+a_{i-1}+2s_{i-1})\,{\mathbb {E}}[{\varvec{C}}_{i-1}(t)] - (\mu _i+\nu _i+\xi _i+s_i-\lambda _i) \, {\mathbb {E}}[{\varvec{C}}_{i}(t)]+\xi _{i+1}\, {\mathbb {E}}[{\varvec{C}}_{i+1}(t)], \quad i \in \{2,\ldots ,N-1\},\nonumber \\ \frac{d\, {\mathbb {E}}[{\varvec{C}}_{N}(t)]}{dt}= & {} (\nu _{N-1}+a_{N-1}+2s_{N-1})\, {\mathbb {E}}[{\varvec{C}}_{N-1}(t)]- (\mu _N+\xi _N-\lambda _N) {\mathbb {E}}[{\varvec{C}}_{N}(t)], \end{aligned}$$

where ${\mathbb {E}}[{\varvec{C}}_i(t)]$ represents the expectation of the random variable ${\varvec{C}}_i(t)$. These equations constitute a homogeneous first-order linear system of ODEs with constant coefficients, which can be written more succinctly in matrix form as follows

2 $$\begin{aligned} \frac{d\textbf{C}(t)}{dt} = \textbf{A} \> \textbf{C}(t), \end{aligned}$$

where

3 $$\begin{aligned} \textbf{C}(t) = \left( \begin{array}{c} {\mathbb {E}}[{\varvec{C}}_{1}(t)]\\ {\mathbb {E}}[{\varvec{C}}_{2}(t)]\\ \vdots \\ {\mathbb {E}}[{\varvec{C}}_{N-1}(t)]\\ {\mathbb {E}}[{\varvec{C}}_{N}(t)]\end{array}\right) ,\quad \textbf{A} = \left( \begin{array}{ccccc} -\Delta _1 &{} \xi _{2} &{} 0 &{} \cdots &{} 0\\ \Lambda _1 &{} -\Delta _2 &{} \xi _{3}&{} \ldots &{}\vdots \\ 0 &{}\ddots &{} \ddots &{}\ddots &{}0\\ \vdots &{} \vdots &{} \Lambda _{N-2} &{} -\Delta _{N-1}&{} \xi _{N}\\ 0 &{} \cdots &{} 0 &{} \Lambda _{N-1} &{}-\Delta _N \end{array} \right) , \end{aligned}$$

and

$$\begin{aligned} \Delta _1= & {} \mu _1+\nu _1+ s_1 -\lambda _1, \\ \Delta _i= & {} \mu _i+\nu _i+s_i+ \xi _{i}-\lambda _i, \quad \Lambda _{i-1} \ =\ \nu _{i-1}+a_{i-1}+2s_{i-1},\ i\in \{2,\ldots ,N-1\}, \\ \Delta _N= & {} \mu _N+ \xi _{N} - \lambda _N,\quad \Lambda _{N-1} \ =\ \nu _{N-1}+a_{N-1}+2s_{N-1}. \end{aligned}$$

The initial value problem of Eq. (2) with $\textbf{C}_0 = \textbf{C}(0)$ has a unique solution [^45^, Theorem 3.9] given by

4 $$\begin{aligned} \textbf{C}(t) = e^{\textbf{A}t}{} \textbf{C}_0, \end{aligned}$$

where $e^{\textbf{A}t}$ represents the matrix exponential

$$\begin{aligned} e^{\textbf{A}t} =\textbf{I}+\textbf{A}t+\textbf{A}^2 \frac{t^2}{2!}+\textbf{A}^3 \frac{t^3}{3!}+ \cdots = \sum _{i=0}^{+\infty }\frac{(\textbf{A}t)^i}{i!}. \end{aligned}$$

The system of equations (2) admits $\lim _{t\rightarrow + \infty }{} \textbf{C}(t)=\textbf{0}_N$ (column vector of zeros) as asymptotic solution. It is exponentially stable (solutions of the system for any initial condition converge to zero) if and only if each eigenvalue of $\textbf{A}$ has a negative real part (see, for example, Ref.[^45^, Corollaries 3.5 and 3.6]).

**Figure 2:**
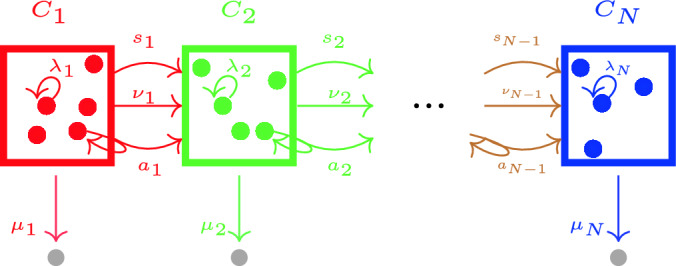
Irreversible stochastic model of cell division, death and forward differentiation for an ordered sequence of compartments. Grey cells represent death events. Self-renewal events occur with per-cell rate $\lambda_i$, symmetric division events with rate $s_i$, and asymmetric division events with rate $a_i$. Differentiation events happen with per-cell rate $\nu_i$. Death events have (per-cell) rate $\mu_i$. Each per-cell rate is a real positive number.

We note that, for certain biological applications, some of the rates in Fig. 1 will be zero, and thus, the analysis of such systems would simplify. For instance, differentiation may be irreversible^9,14^, so that $\xi _i=0$ for $i\in \{2,\ldots ,N\}$ as shown in Fig. 2. We will refer to this scenario as the *irreversible model*. In this case, and if one considers a single progenitor cell starting in compartment $C_1$ at time $t=0$, $\textbf{C}(0)=(1,0,\ldots ,0)^T$, it is possible to obtain the mean number of cells for any compartment. In fact, one has

5 $$\begin{aligned} {\mathbb {E}}[{\varvec{C}}_{i}(t)]= & {} \left\{ \begin{array}{ll} e^{-\Delta _1t}, &{} i=1,\\ \left( \prod \limits _{l=1}^{i-1} \Lambda _l \right) \sum \limits _{j=1}^i e^{-\Delta _j t} \prod \limits _{\begin{array}{c} m=1 \\ m\ne j \end{array}}^i (\Delta _m-\Delta _j)^{-1},&i\in \{2,\ldots ,N\}. \end{array}\right. \end{aligned}$$

Equation (5) is well-defined if $\Delta _i\ne \Delta _j$ for pairs (*i*, *j*) with $i \ne j$. If this is not the case, alternative analytic solutions can be found. For example, if $\Delta _i=\Delta _j$ for all $i,j\in \{1,\ldots ,N\}$, Eq. (5) simplifies to

6 $$\begin{aligned} {\mathbb {E}}[{\varvec{C}}_{i}(t)] = \left( \prod \limits _{l=1}^{i-1} \Lambda _l \right) \frac{t^{i-1}}{(i-1)!}e^{-\Delta _it},\quad t\ge 0. \end{aligned}$$

We note that this is consistent with existing results in the literature. In particular, the most general solution for the case of repeated eigenvalues can be found in Ref.^46^, which analysed the radioactive decay of a chain of nuclides. It is clear that in the irreversible model $\lim _{t\rightarrow +\infty }{\mathbb {E}}[{\varvec{C}}_i(t)]=0$, when $\Delta _i>0$ $\forall i\in \{1,\ldots ,N\}$. This is consistent since $\{-\Delta _i:\ i\in \{1,\ldots ,N\}\}$ are the eigenvalues of $\textbf{A}$ in this case.

For biological applications^3,14^ such as those considered in Section “Results”, it is of interest to determine the cumulative average number of cells that arrive to the final compartment, $C_N$, starting with a single “progenitor” or precursor cell in compartment $C_1$. To this end, in the irreversible model one can set $\lambda _N=\mu _N=0$, so that cells arriving into $C_N$ accumulate and can be counted. Then, for the last compartment $\Delta _N=0$ and thus, Eq. (5) leads to

7 $$\begin{aligned} {\mathbb {E}}[{\varvec{C}}_{N}(t)] = \left( \prod \limits _{l=1}^{N-1} \Lambda _l \right) \left[ \sum \limits _{j=1}^{N-1} e^{-\Delta _j t} \prod \limits _{\begin{array}{c} m=1 \\ m\ne j \end{array}}^{N-1} (\Delta _m-\Delta _j)^{-1} + \prod \limits _{\begin{array}{c} m=1 \end{array}}^{N-1} \Delta _m^{-1} \right] , \end{aligned}$$

which is well-defined if $\Delta _i\ne \Delta _j$ for all $i,j\in \{1,\ldots ,N-1\}$. From the previous equation, we have

8 $$\begin{aligned} \lim _{t\rightarrow +\infty } {\mathbb {E}}[ {\varvec{C}}_{N}(t)]=\prod _{i=1}^{N-1}\frac{\Lambda _i}{\Delta _i}. \end{aligned}$$

Interestingly, this limit also holds if $\Delta _i=\Delta _j$ for all $i,j \in \{1,\ldots ,N-1\}$ and $\Delta _N=0$. In this case one can write

9 $$\begin{aligned} {\mathbb {E}}[{\varvec{C}}_{N}(t)]= & {} \prod _{i=1}^{N-1}\frac{\Lambda _i}{\Delta _i} -\sum _{j=1}^{N-1} {\mathbb {E}}[{\varvec{C}}_j(t)] \prod _{i=j}^{N-1}\frac{\Lambda _i}{\Delta _i}. \end{aligned}$$

Under population extinction conditions (that is, when $\Delta _i>0$ for all $i\in \{1,\ldots ,N-1\}$, so that the population of cells in intermediate compartments, $\{1,\ldots ,N-1\}$, dies out and only “exiting” cells remain in $C_N$ at late times), $\lim _{t\rightarrow +\infty } {\mathbb {E}}[ {\varvec{C}}_{i}(t)]=0$ for $i\in \{1,\ldots ,N-1\}$, and thus Eq. (8) also holds.

A particular feature of this system is that cells behave independently from each other. This means that the dynamics of the progeny of a set of *M* progenitor (or precursor) cells in compartment $C_1$ at time $t=0$, can be analysed as *M* independent stochastic processes. Thus, in Section “The progeny of a single progenitor cell” we consider a number of summary statistics of interest related to the stochastic journeys of cell progenies from a single cell starting at a given compartment (typically compartment $C_1$).

### The progeny of a single progenitor cell

**Figure 3:**
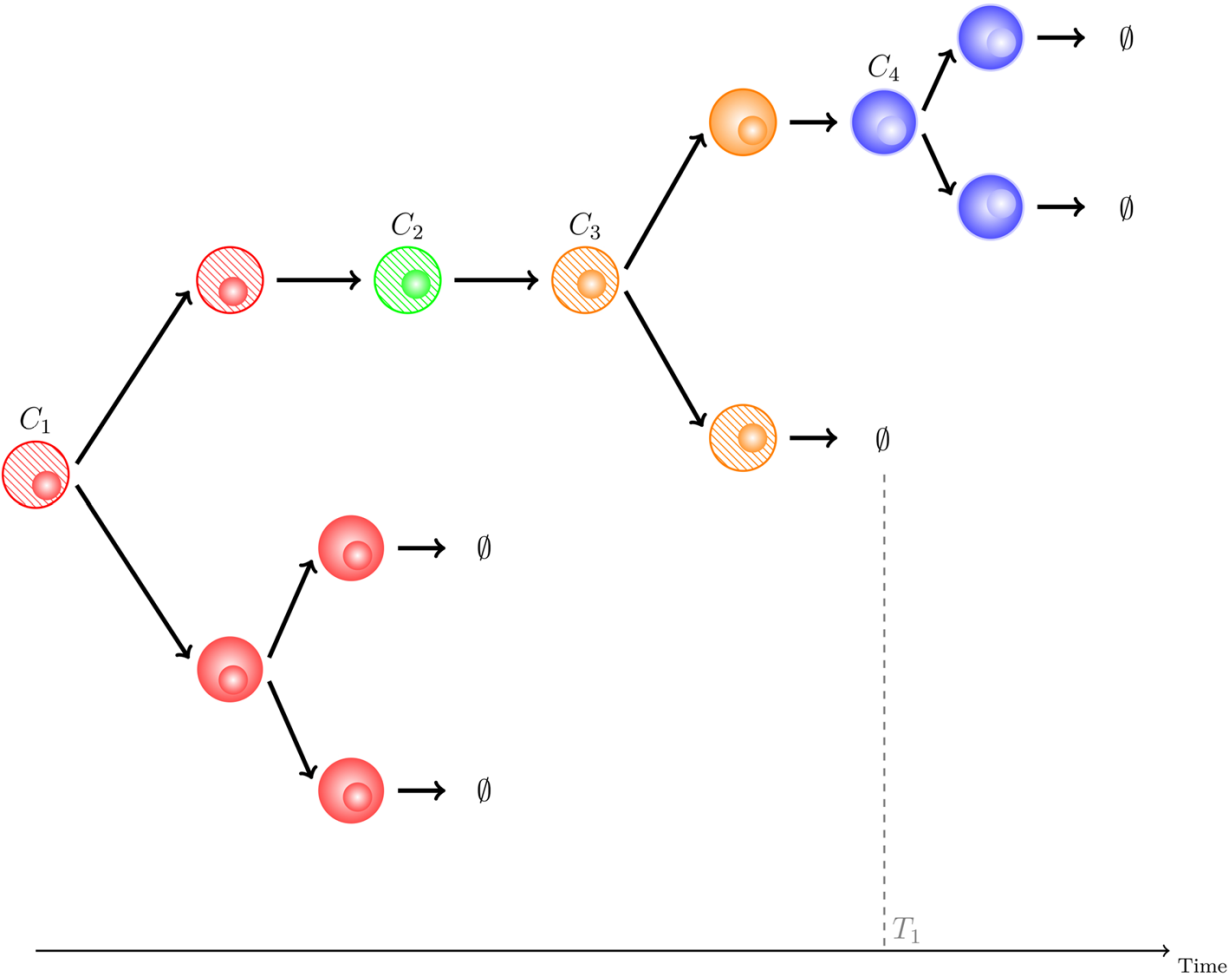
A realisation of the stochastic process tracking the progeny of a single progenitor cell which starts in compartment $C_1$. The cell tracked (see Section “Lifeline analysis”) is shown as striped. Whenever the tracked cell divides, we follow one of the daughter cells selected at random (see division in $C_3$). For each cell, the colour indicates the compartment where it is at any given time. Here, the tracked cell dies in $C_3$ (brown), while its progeny continues up to $C_4$. In this example, $G_1 = 8 = G_1(1)+G_1(2)+G_1(3)+G_1(4)=4+0+2+2$.

For a cell starting in compartment $C_i$, we can define $G_i$ to be the random variable representing the total number of cells in the progeny of this cell. Cells in the progeny are the daughters, granddaughters, etc., of the progenitor (or precursor) cell, which originate from division events (either self-renewal, asymmetric or symmetric) in any compartment over time, not including the progenitor cell itself. It is a summary statistic of the process which quantifies the proliferative potential of a single cell in compartment $C_i$, and its offspring. For example, in Fig. 3, we represent a particular realisation of the stochastic process with $G_1=8$.

The mean number of cells in the progeny of a progenitor cell, $m_i={\mathbb {E}}[G_i]$, for any initial compartment of interest $i\in \{1,\ldots ,N\}$, can be obtained with first-step arguments by conditioning on the next event that occurs in the stochastic process. This approach leads to the following system of equations

$$\begin{aligned} &\Delta _1m_1= {} \Lambda _1m_2 + 2(\lambda _1+a_1+s_1), \\& \Delta _im_i= {} \Lambda _i m_{i+1} +\xi _{i} m_{i-1} +2(\lambda _i+a_i+s_i),\quad i\in \{2,\ldots ,N-1\}, \\& \Delta _Nm_N= {} \xi _N m_{N-1} + 2\lambda _N. \end{aligned}$$

The system above can be expressed in matrix form with the column vectors $\textbf{m}=\left( m_1, \ldots , m_N \right) ^T$ and $\textbf{b}=\left( 2(\lambda _1+a_1+s_1), \ldots ,\right.$ $\left. 2(\lambda _{N-1}+a_{N-1}+s_{N-1}),\right.$ $\left. 2\lambda _N\right) ^T$, as follows

10 $$\begin{aligned} \textbf{J}\, \textbf{m} = \textbf{b}, \end{aligned}$$

with a tri-diagonal coefficient matrix

11 $$\begin{aligned} \textbf{J}= \left( \begin{array}{cccccc} \Delta _1&{}- \Lambda _1 &{} 0 &{} 0 &{} \cdots &{} 0\\ - \xi _{2} &{} \Delta _2 &{} -\Lambda _2&{} 0&{} \cdots &{}0\\ 0 &{} -\xi _3 &{} \Delta _3&{} -\Lambda _3&{} \cdots &{}0\\ \vdots &{}\ddots &{}\ddots &{} \ddots &{}\ddots &{} \vdots \\ 0&{} \cdots &{} 0 &{}- \xi _{N-1} &{} \Delta _{N-1} &{} -\Lambda _{N-1}\\ 0 &{} \cdots &{} 0 &{}0 &{} - \xi _{N} &{} \Delta _{N} \end{array} \right) . \end{aligned}$$

One can exploit the tri-diagonal structure of $\textbf{J}$ to obtain an explicit, or recursive, solution. In particular, by following a Gaussian forward-elimination backward-substitution approach, such as the Thomas algorithm^47,48^, one can obtain the recursive equations

12 $$\begin{aligned} m_N= & {} \rho _N,\quad m_i \ =\ \rho _i - \gamma _i m_{i+1},\quad i\in \{1,\ldots ,N-1\}, \end{aligned}$$

where $$\gamma _1 = -\Delta ^{-1}_1 \Lambda _1$$, $$\rho _1 = 2\Delta ^{-1}_1(\lambda _1+s_1+a_1)$$, and

$$\begin{aligned} \gamma _i= & {} -\frac{\Lambda _i}{\Delta _i+\xi _{i} \gamma _{i-1} },\quad i \in \{2,\ldots ,N-1\}, \\ \rho _i= & {} \frac{2(\lambda _i+s_i+a_i) + \xi _{i} \rho _{i-1}}{\Delta _i+\xi _{i} \gamma _{i-1}},\quad i\in \{2,\ldots ,N\}. \end{aligned}$$

Using backward-substitution, this recursive scheme leads to the explicit solution

13 $$\begin{aligned} m_i= & {} \sum _{j=i}^N\, (-1)^{j-i}\rho _j \, \left( \prod _{l=i}^{j-1} \gamma _l \right) ,\quad i\in \{1,\ldots ,N\}, \end{aligned}$$

where $\prod _{l=i}^{i-1} \gamma _l = 1$. A condition on the parameters arises during the implementation of the recursive scheme

$$\begin{aligned} \Delta _1>0,\quad \Delta _i+\xi _i\gamma _{i-1}> & {} 0,\quad i\in \{2,\ldots ,N\}, \end{aligned}$$

so that the mean values $m_1,\ldots ,m_N$ are finite and non-negative, for all $i\in \{1,\ldots ,N\}$. This ensures that the number of cells in the progeny of a progenitor cell is finite with probability one, ${\mathbb {P}}(G_i<+\infty )=1$, since $m_i={\mathbb {E}}[G_i]={\mathbb {E}}[G_i \vert G_i<+\infty ]{\mathbb {P}}(G_i<+\infty )+{\mathbb {E}}[G_i \vert G_i=+\infty ]{\mathbb {P}}(G_i=+\infty )$. In the irreversible case, where $\xi _i=0$ for $i\in \{2,\ldots ,N\}$, the solution above simplifies to

14 $$\begin{aligned} m_i=2\sum ^{N}_{j=i} \frac{\lambda _j+a_j+s_j}{\Delta _{j}}\left( \prod _{l=i}^{j-1} \frac{\Lambda _l}{\Delta _l}\right) ,\quad i\in \{1,\ldots ,N\}, \end{aligned}$$

where for $j=N$, we set $a_N=s_N=0$. For $i=N$, the empty product above is equal to one, so that $m_N=\frac{2\lambda _N}{\mu _N-\lambda _N}$. For the irreversible model the condition to have finite and non-negative solutions becomes $\Delta _i>0$ for all $i\in \{1,\ldots ,N\}$. Direct inspection of Fig. 2 shows that the condition $\Delta _i>0$ avoids unlimited accumulation of cells in compartment $C_i$.

#### Probability generating function

Let us now go beyond the mean number of cells. For the irreversible model we can consider the probability generating function of $G_i$,

$$\begin{aligned} \Phi _i(z)={\mathbb {E}}(z^{G_i})=\sum _{k=0}^{+\infty }{\mathbb {P}}(G_i=k) z^k. \end{aligned}$$

The variable $G_i$ counts the cells in the progeny of a progenitor cell starting in compartment $C_i$, arising from division events (self-renewal, asymmetric and symmetric division), not including the progenitor cell itself. To include the progenitor cell, one can define $S_i \equiv G_i+1$, and denote the new generating function by $\Psi _i(z)={\mathbb {E}}(z^{S_i})$. The total expectation law over all possible first events implies that

15 $$\begin{aligned} {\mathbb {E}}(z^{S_i}) = \sum _{E_j} {\mathbb {E}}(z^{S_i} \> \vert \> \text {event} \> E_j){\mathbb {P}}(E_j), \end{aligned}$$

with $E_j\in \{$death, differentiation, self-renewal, asymmetric division, symmetric division$\}$. This leads to

16 $$\begin{aligned} \Psi _i(z)= & {} \frac{\mu _i}{\Sigma _i}z + \frac{\nu _i}{\Sigma _i}z \Psi _{i+1}(z) + \frac{\lambda _i}{\Sigma _i} z (\Psi _i(z))^2 + \frac{a_i}{\Sigma _i}z \Psi _i(z) \Psi _{i+1}(z)+\frac{s_i}{\Sigma _i}z (\Psi _{i+1}(z))^2, \end{aligned}$$

with $\Sigma _i=\mu _i+\nu _i+\lambda _i+a_i+s_i$. Since $S_i=G_i+1$, one can write $z \Phi _i(z) = \Psi _i(z),$ so that

17 $$\begin{aligned} \lambda _i z^2 \Phi _{i}^2(z) + (a_i z^2 \Phi _{i+1}(z) -\Sigma _i) \Phi _{i}(z) + \Phi _{i+1}(z)(\nu _iz +s_i z^2 \Phi _{i+1}(z))+ \mu _i\ =\ 0. \end{aligned}$$

The probability generating functions above agree with the mean values obtained earlier. In particular, by differentiating with respect to *z*, and setting $z=1$, we have

18 $$\begin{aligned} (\Sigma _i-a_i-2\lambda _i) \Phi _{i}'(1) = 2(a_i+\lambda _i+s_i) + (\nu _i +a_i+2s_i) \Phi _{i+1}'(1), \end{aligned}$$

which can be solved recursively, leading to

19 $$\begin{aligned} {\mathbb {E}}(G_i) = \Phi _{i}'(1)&= 2 \sum _{j=i}^N \frac{a_j+\lambda _j+s_j}{\Delta _j} \left( \prod _{l=i}^{j-1} \frac{\Lambda _l}{\Delta _l} \right) , \end{aligned}$$

in agreement with Eq. (14). We also note that when $i=N$, the cells in the progeny of a single progenitor cell in compartment $C_N$ arise from a linear birth-and-death process, which in discrete time has death probability, $p_d=\frac{\mu _N}{\mu _N+\lambda _N}$, and birth probability, $p_b=\frac{\lambda _N}{\mu _N+\lambda _N}$. Then, a first-step argument for the random variable $S_N$ leads to

$$\begin{aligned} \Psi _N(z)= & {} p_d z + p_b z \Psi _N^2(z) \ =\ z \phi _N(\Psi _N(z)), \end{aligned}$$

with $\phi _N(z)=p_d+ p_b z^2$. We can then write

$$\begin{aligned} \Phi _N(z)=\frac{\Psi _N(z)}{z}= \frac{z \> \phi _N(\Psi _N(z))}{z} = \phi _N(z \> \Phi _N(z)), \end{aligned}$$

which has solution

20 $$\begin{aligned} \Phi _N(z) = p_d\frac{1-\sqrt{1-4x}}{2x}\quad \text {where}\quad x=p_dp_bz^2. \end{aligned}$$

We find ${\mathbb {P}}(G_N=k)$ as the coefficient of the power of $z^k$, using the property of the Catalan numbers^49^. In particular, since

$$\begin{aligned} {\sum _{n=0}^{\infty }C_nx^n = \frac{1-\sqrt{1-4x}}{2x}}, \end{aligned}$$

we obtain

21 $$\begin{aligned} \begin{aligned} {\Phi _N(z)}&{= p_d\sum _{n=0}^{\infty }C_n(p_dp_b)^nz^{2n}= \sum _{n=0}^{\infty }\frac{(2n)!}{(n+1)!n!}p_d^{n+1}p_b^nz^{2n}= p_d + z^2p_d^2p_b + z^42p_d^3p_b^2 + z^65p_d^3p_b^4 + \cdots .} \end{aligned} \end{aligned}$$

Note that ${\mathbb {P}}(G_N=k+2)/{\mathbb {P}}(G_N=k) = \frac{4k}{k+3}p_dp_b$. Assuming $\lambda _N<\mu _N$, we verify that ${\mathbb {E}}(G_N) = \Phi _N'(1) = \frac{2\lambda _N}{\mu _N-\lambda _N}$.

#### Compartmental analysis of the progeny

Cells in the progeny of a single progenitor can belong to different compartments, as shown in Fig. 3. For a progenitor cell starting in compartment $C_i$, compartments $C_j$, $j\in \{1,2,\ldots ,N\}$ with greater proliferative potential will contribute more to $G_i$. The proliferative potential of compartment *j* depends on the parameters $\lambda _j,a_j,s_j,\mu _j,\nu _j,\xi _j$, but also on the number of cells arriving into that compartment. It is of interest then to write $G_i=\sum _{j=1}^NG_i(j)$, with $G_i(j)$ the number of cells in compartment $C_j$ which belong to the progeny of the progenitor cell from compartment $C_i$. For example, for the stochastic realisation of Fig. 3, $G_1=G_1(1)+G_1(2)+G_1(3)+G_1(4)=4+0+2+2=8$.

One can follow similar arguments to the ones in Section “The progeny of a single progenitor cell” to compute the mean quantities $m_i(j)\equiv {\mathbb {E}}[G_i(j)]$. In particular, for an initial compartment $C_i$, a first-step argument yields the following equations

$$\begin{aligned} & \Delta _i m_{i}(i)= {} 2 \lambda _i + a_i +\Lambda _i\, m_{i+1}(i) +\xi _i m_{i-1}(i), \\& \Delta _im_{i}(i+1)= {} 2 s_i + a_i +\Lambda _i\, m_{i+1}(i+1) +\xi _{i}m_{i-1}(i+1), \\& \Delta _im_{i}(j)= {} \Lambda _im_{i+1}(j) +\xi _{i} m_{i-1}(j),\quad j\notin \{i,i+1\}, \end{aligned}$$

where we implicitly set $\xi _{1} = 0$, and $\forall j \in \{1, 2, \ldots , N\}$, $m_{N+1}(j)=m_N(N+1)=m_0(j) = 0$ for notational convenience. Making use of a recursive approach one can show that for any $j\in \{1,\ldots ,N\}$, we have

$$\begin{aligned} m_N(j)= & {} \rho _N(j),\quad m_i(j) \ =\ \rho _i(j) - \gamma _i m_{i+1}(j),\quad i\in \{1,\ldots ,N-1\}, \end{aligned}$$

where $$\gamma _1 = -\Delta ^{-1}_1 \Lambda _1$$, $$\rho _1(j) = \Delta ^{-1}_1 d_{(1,j)}$$, and

$$\begin{aligned} \gamma _i= & {} -\frac{\Lambda _i}{\Delta _i+\xi _{i} \gamma _{i-1} },\quad \quad i \in \{2,\ldots ,N-1\}, \\ \rho _i(j)= & {} \frac{d_{(i,j)}+ \xi _{i} \rho _{i-1}(j)}{\Delta _i+\xi _{i} \gamma _{i-1}},\quad i,j\in \{2,\ldots ,N\}, \end{aligned}$$

with

$$\begin{aligned} d_{(i,j)}= & {} {\left\{ \begin{array}{ll} 2\lambda _i + a_i, &{} \quad \text {if} \quad j=i, \\ 2s_i+a_i, &{} \quad \text {if} \quad j=i+1,\\ 0, &{} \quad \text {otherwise.} \end{array}\right. } \end{aligned}$$

This recursive scheme leads to the solution

22 $$\begin{aligned} m_{i}(j) =\sum _{k=i}^N(-1)^{k-i}\rho _ k(j)\, \left( \prod _{p=i}^{k-1} \gamma _p \right) , \end{aligned}$$

where $\prod _{p=i}^{i-1} \gamma _p = 1$. This expression simplifies further for the irreversible model, since $\xi _i=0$ for all $i\in \{2,\ldots ,N\}$. In this instance, $m_i(j)=0$ whenever $i>j$, and we can write

$$\begin{aligned} m_{i}(i)= & {} \Delta _i^{-1} (2 \lambda _i + a_i ),\\ m_{i}(j)= & {} \Delta ^{-1}_{j-1} \left( \prod _{p=i}^{j-2}\Delta ^{-1}_p \Lambda _p\right) \left( d_{(j-1,j)}+ d_{(j,j)}\Delta ^{-1}_{j} \Lambda _{j-1}\right) ,\quad j\in \{i+1, \ldots ,N\}, \end{aligned}$$

for any $i\in \{1,\ldots ,N-1\}$. Finally, we note that $\prod _{p=i}^{i-1}\Delta ^{-1}_p \Lambda _p=1$, and $m_{N}(N) = \frac{2\lambda _N}{\Delta _N}$.

### Lifeline analysis

**Figure 4:**
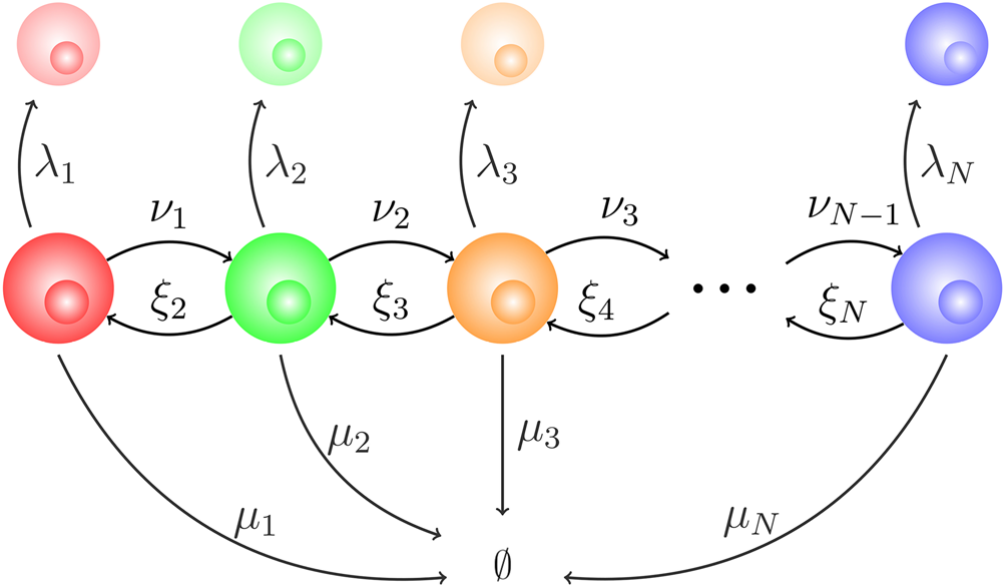
Representation of the process ${{{\mathcal {Y}}}}$ to follow the fate of a single cell.

In the previous sections we have analysed several summary statistics of the progeny of a progenitor cell. This section is devoted to summary statistics related to the history of a tracked cell, extending the analysis proposed in Ref.^42^, in the situation when all cell divisions are self-renewals; that is, $s_i=a_i=0$ for all $i\in \{1,\ldots ,N\}$. We construct lifelines containing a single cell at any time, starting in compartment $C_i$. The line continues when that cell divides (by selecting one daughter cell at random) and terminates when the cell dies. In any given compartment, this cell can divide, move to another compartment, or die. Since the only type of division is self-renewal, we use the theoretical “trick” of identifying one of the daughter cells as a continuation of the mother, and the other as a new cell. The dynamics can then be represented by the CTMC ${{{\mathcal {Y}}}}=\{Y(t):\ t\ge 0\}$ over the state space ${{{\mathcal {S}}}}=\{ C_1, C_2,\ldots , C_N,\emptyset \}$, where *Y*(*t*) represents the state of the cell at time *t*; the cell is either in some compartment, $C_j$, or it has died (state $\emptyset$). A schematic representation of the process ${{{\mathcal {Y}}}}$ is given in Fig. 4, and a particular realisation of this stochastic process can be seen in Fig. 3, where the tracked cell is shown with stripes.

We study the stochastic process with the following summary statistics:

- The duration, $T_i$, of the lifeline, starting in compartment $C_i$,
  $$\begin{aligned} T_i= & {} \inf \{t\ge 0:\ Y(t)=\emptyset \vert Y(0)=C_i\}, \end{aligned}$$
  which measures the survival potential of cells in the system depending on their initial compartment,
- The number of divisions along a path through the family tree, $D_i$, which quantifies the proliferative capacity of cells according to their initial compartment, and
- The probability that the lifeline ends in a compartment $C_j$; that is, $\beta _i(j)={\mathbb {P}}(Y(T_i-\Delta t)=C_j)$ for small enough $\Delta t$.

#### Lifespan of a tracked cell

Let $\tau _i={\mathbb {E}}[T_i]$ be the mean lifespan of a cell starting in compartment $C_i$. By conditioning on the events the cell can undergo in the initial compartment, we obtain the following recursive equations

$$\begin{aligned} &(\mu _1+\nu _1) \tau _{1}= {} \nu _1 \tau _{2} +1,\\& (\mu _i+\nu _i+\xi _i) \tau _{i}= {} \nu _i \tau _{i+1} + \xi _i \tau _{i-1} + 1,\quad i\in \{1,\ldots ,N-1\},\\& (\mu _N+\xi _N) \tau _{N}= {} \xi _N \tau _{N-1} + 1. \end{aligned}$$

The previous equations have the general solution

$$\begin{aligned} \tau _i =\sum _{k=i}^N(-1)^{k-i}{{\bar{\rho }}}_ k\, \left( \prod _{p=i}^{k-1} {{\bar{\gamma }}}_p \right) , \end{aligned}$$

where $\prod _{p=i}^{i-1} {{\bar{\gamma }}}_p = 1$. We have made use of the notation ${{\bar{\gamma }}}_1 = -{{\bar{\Delta }}}^{-1}_1 \nu _1$, ${{\bar{\rho }}}_1 = {{\bar{\Delta }}}^{-1}_1$, with

$$\begin{aligned} {{\bar{\gamma }}}_i= & {} -\frac{\nu _i}{{{\bar{\Delta }}}_i+\xi _{i} {{\bar{\gamma }}}_{i-1} },\quad i \in \{2,\ldots ,N-1\}, \\ {{\bar{\rho }}}_i= & {} \frac{1+ \xi _{i} {{\bar{\rho }}}_{i-1}}{{{\bar{\Delta }}}_i+\xi _{i} {{\bar{\gamma }}}_{i-1}},\quad i\in \{2,\ldots ,N\}, \end{aligned}$$

where ${{\bar{\Delta }}}_i = \mu _i + \nu _i +\xi _i$. A simpler solution is obtained in the irreversible model

$$\begin{aligned} \tau _i =\sum _{k=i}^N \frac{1}{\mu _{k}+\nu _{k}} \, \left( \prod _{p=i}^{k-1} \frac{\nu _{p}}{\mu _{p}+\nu _p} \right) ,\quad i\in \{1,\ldots ,N\}, \end{aligned}$$

where $\prod _{p=i}^{i-1} \frac{\nu _{p}}{\mu _{p}+\nu _p} = 1$, and we set $\nu _N=0$ for notational convenience.

A similar approach allows one to compute the Laplace-Stieltjes transform of $T_i$, and any of its higher order moments^50^. In particular, one could obtain a system of equations, via first-step arguments, for the Laplace-Stieltjes transform of $T_i$, $\phi _i(z)={\mathbb {E}}[e^{-zT_i}]$. The different order moments can be computed via successive differentiation of the transform, while the probability density function of $T_i$ can be computed via its numerical inversion^51^. For example, in the irreversible model, the second-order moment of the lifespan of a cell starting in compartment $C_i$ is given by

$$\begin{aligned} {\mathbb {E}} [T_{i}^2] = \sum ^{N}_{j=i} R_j \left( \prod _{r=i}^{j-1}\frac{\nu _r}{\mu _r+\nu _r} \right) ,\quad i\in \{1,\ldots ,N-1\}, \end{aligned}$$

where $\prod _{p=i}^{i-1} \frac{\nu _r}{\mu _r+\nu _r} = 1$, $R_i= \frac{2(\nu _i \tau _{i+1}+1)}{(\mu _i+\nu _i)^2 }$, and $R_N = \frac{2}{\mu _N^2}$. We note that if the cell starts in the last compartment $C_N$, ${\mathbb {E}} [T_N^2]=2\mu _N^{-2}$, since $T_N\sim \text {Exp}(\mu _N)$.

#### Number of divisions along a lifeline

Let us define $D_i$, the number of division events along the history of the tracked cell, starting in compartment $C_i$. We compute the average value, $\eta _i={\mathbb {E}}[D_i]$, with the first-step formula

$$\begin{aligned} & (\lambda _1+\mu _1+\nu _1)\eta _1= {} \lambda _1(\eta _1+1)+\nu _1\eta _{2},\\& (\lambda _i+\mu _i+\nu _i+\xi _i)\eta _i= {} \lambda _i(\eta _i+1)+\nu _i\eta _{i+1}+\xi _i\eta _{i-1},\quad i\in \{1,\ldots ,N-1\},\\& (\lambda _N+\mu _N+\xi _N)\eta _N= {} \lambda _N(\eta _N+1)+\xi _N\eta _{N-1}. \end{aligned}$$

The previous equations have solution

23 $$\begin{aligned} \eta _i =\sum _{k=i}^N(-1)^{k-i}{{{\tilde{\rho }}}}_ k\, \left( \prod _{p=i}^{k-1} {{\bar{\gamma }}}_p \right) , \end{aligned}$$

where $$\prod _{p=i}^{i-1} {{\bar{\gamma }}}_p = 1$$, $${{\tilde{\rho }}}_1 = {{\bar{\Delta }}}^{-1}_1 \lambda _1$$, and

$$\begin{aligned} {{\tilde{\rho }}}_i= & {} \frac{\lambda _i+ \xi _{i} {{\tilde{\rho }}}_{i-1}}{{{\bar{\Delta }}}_i+\xi _{i} {{\bar{\gamma }}}_{i-1}},\quad i\in \{2,\ldots ,N\}, \end{aligned}$$

with ${{\bar{\Delta }}}_i$ and ${{\bar{\gamma }}}_i$ defined as above. In the irreversible model, this expression simplifies to

$$\begin{aligned} {\eta _i }=\sum _{k=i}^N \frac{\lambda _k}{\mu _{k}+\nu _{k}} \, \left( \prod _{p=i}^{k-1} \frac{\nu _{p}}{\mu _{p}+\nu _p} \right) ,\quad i\in \{1,\ldots ,N-1\}, \end{aligned}$$

where $\prod _{p=i}^{i-1} \frac{\nu _{p}}{\mu _{p}+\nu _p} = 1$. We note that in this case $\eta _N=\lambda _N\mu _N^{-1}$, since $D_N\sim \text {Geometric} \left( \frac{\lambda _N}{\mu _N+\lambda _N}\right)$.

A division event may occur at any instant along the lifeline of the cell, which may visit different compartments over time. Thus, one can determine the proliferative potential of the cell during its (eventual) visit to each compartment by considering $D_i=\sum _{j=1}^ND_i(j)$, where $D_i(j)$ is the number of divisions which occur exactly in compartment $C_j$. Average values, $\eta _i(j)\equiv {\mathbb {E}}[D_i(j)]$, can be computed (again) with first-step arguments. For instance, in the irreversible model, one has

$$\begin{aligned} \eta _i(i)= & {} \frac{\lambda _i}{\mu _i + \nu _i}, \\ \eta _{i}(j)= & {} \frac{\lambda _j}{\mu _j+\nu _j}\left( \prod _{k=i}^{j-1} \frac{\nu _{k}}{\mu _k+\nu _k}\right) ,\quad j \ge i+1, \end{aligned}$$

for $i\in \{1, \ldots , N\}$ and $\eta _N(N+1)=0$. We note that the expression above is consistent with the interpretation that, in the irreversible model, $D_i(j)\sim \text {Geometric}\left( \frac{\lambda _j}{\lambda _j+\nu _j+\mu _j}\right)$ restricted to the arrival of the cell to compartment $C_j$. In general, one can write

$$\begin{aligned} \eta _i(j)= & {} {\mathbb {E}}[D_i(j)]={\mathbb {E}}[D_i(j)\ \vert \ {cell\;ever\;visits }~ C_j]\>{\mathbb {P}}(cell\;ever\;visits ~ C_j)+{\mathbb {E}}[D_i(j)\ \vert \ cell\;never\;visits ~ C_j]\>{\mathbb {P}}(cell\;never\;visits ~ C_j), \end{aligned}$$

and, since ${\mathbb {E}}[D_i(j)\ \vert \ {cell\;never\;visits} ~ C_j]=0$, the quantity $\eta _i(j)$ accounts for the probability of the cell not visiting this compartment.

More generally, we consider the complete probability distribution of $D_i$, defined by $\omega _i(n) \equiv {\mathbb {P}}(D_i =n)$, the probability that a single cell starting in compartment $C_i$ divides exactly *n* times before it dies or leaves the system, for any non-negative integer *n*. One can show that

$$\begin{aligned} \omega _{i}(n)= & {} \sum ^{N}_{k=i} (-1)^{k-i}{{\hat{\rho }}}_k(n) \left( \prod _{j=i}^{k-1} {{\hat{\gamma }}}_j\right) , \quad i\in \{1,\ldots ,N\}, \,\, {n =0,1,2,3, \ldots }, \end{aligned}$$

where $\prod _{j=i}^{i-1} {{\hat{\gamma }}}_j= 1$. We have introduced the notation ${{\hat{\rho }}}_1(n) = (\lambda _1 \omega _1(n-1)+ \mu _1 1_{n=0}){{\hat{\Delta }}}_1^{-1}$, ${{\hat{\gamma }}}_1 = -\nu _1 {{\hat{\Delta }}}_1^{-1}$, and

$$\begin{aligned} {{\hat{\rho }}}_i(n) = \frac{\lambda _i \omega _i(n-1) +\mu _i 1_{n=0}+ \xi _i {{\hat{\rho }}}_{i-1}(n)}{{{\hat{\Delta }}}_i+\xi _i {{\hat{\gamma }}}_{i-1}},\quad {{\hat{\gamma }}}_i = \frac{-\nu _i}{{{\hat{\Delta }}}_i+\xi _i {{\hat{\gamma }}}_{i-1}}, \end{aligned}$$

with ${{\hat{\Delta }}}_i = \mu _i + \lambda _i + \xi _i + \nu _i$. We note that $\omega _i(-1) = 0$ and that $1_{n=0}$ is the indicator function, equal to 1 if $n=0$, and zero otherwise. Thus, the probabilities $\omega _i(n)$ can be computed recursively for increasing values of *n*, since $\omega _i(n)$ depends on $\omega _i(n-1)$.

#### Cell death

The probability that the tracked cell dies in compartment *j* is denoted by

$$\begin{aligned} \beta _{i}(j)= & {} {\mathbb {P}}(Y(T_i-\Delta t)=C_j), \end{aligned}$$

for small enough $\Delta t$, and for any $i,j\in \{1,\ldots ,N\}$. Once again, a first-step argument leads to the following recursive relations

$$\begin{aligned}& (\mu _1+\nu _1)\beta _{1}(j)= \nu _1 \beta _{2}(j)+\mu _1 1_{j=1}, \\& (\mu _i+\nu _i+\xi _i)\beta _{i}(j)= \nu _i \beta _{i+1}(j)+\xi _i \beta _{i-1}(j)+\mu _i 1_{i=j},\\ &(\mu _N+\xi _N)\beta _{N}(j)= \xi _N \beta _{N-1}(j)+\mu _N 1_{j=N}, \end{aligned}$$

with solution

$$\begin{aligned} \beta _{i}(j)= & {} \sum ^{N}_{k=i} (-1)^{k-i} \bar{\rho }_k(j) \left( \prod _{p=i}^{k-1} \bar{\gamma }_p\right) \quad i,j\in \{1,\ldots ,N\}, \end{aligned}$$

where $\bar{\rho }_1(j) = \mu _1 \bar{\Delta }_1^{-1} 1_{j=1}$ for $j\in \{1,\ldots ,N\}$, and

$$\begin{aligned} \bar{\rho }_i(j) = \frac{\mu _i 1_{i=j} +\xi _i \bar{\rho }_{i-1}(j)}{\bar{\Delta }_i +\xi _i\bar{\gamma }_{i-1}},\quad i\in \{2, \ldots , N\},\ j\in \{1, \ldots ,N\}. \end{aligned}$$

For the irreversible model, for $i\in \{1,\ldots ,N\}$, we have

$$\begin{aligned} \beta _{i}(i)= & {} \frac{\mu _i}{\mu _{i} + \nu _{i}},\quad \beta _{i}(j) \ =\ \frac{\mu _j}{\mu _j+\nu _j}\prod _{k=i}^{j-1} \frac{\nu _{k}}{\mu _k+\nu _k}, \quad j\in \{i+1, \ldots ,N\}. \end{aligned}$$

We conclude this analysis with a comment. A particular advantage of obtaining analytical expressions for the summary statistics is that they allow one to explicitly compute sensitivities, $\partial \beta _i(j)/\partial \theta$, or elasticities, $(\partial \beta _i(j)/\partial \theta )/(\beta _i(j)/\theta$), with respect to model parameters of interest, $\theta$. This can be rather useful when considering a complex model (with many parameters), as illustrated in Section ’Tracking a thymocyte during its development”. A local sensitivity analysis of this kind provides a quantification of the impact that small perturbations of model parameters can have on a given summary statistics of interest, and is particularly relevant when a subset of the model parameters are being estimated from experimental data sets, and thus, will carry inherent uncertainties. Finally, we refer the reader to the Supplementary Material for extra details on different calculations related to this section.

## Results

We illustrate our approach with three case studies; in Section “Asymmetric and symmetric division: the case of four compartments”, we implement the methods from Sections “Mean number of cells in each compartment” and “The progeny of a single progenitor cell” to explore the impact of asymmetric and symmetric division events, for the specific case of $N=4$ compartments. We perform sensitivity analysis for the probabilities of self-renewal, asymmetric and symmetric division. The impact of asymmetric and symmetric division is further analysed in Section “Hematopoietic stem cells: self-renewal, asymmetric and symmetric division”, where we consider hematopoietic stem cells, in light of recent experimental data and a mathematical model proposed in Ref.^9^. Finally, in Section “Tracking a thymocyte during its development” we apply the analysis from Section “Lifeline analysis” to an existing model of thymic T cell development^14^.

### Asymmetric and symmetric division: the case of four compartments

Let us consider the case $N=4$, for illustrative purposes, where the last compartment, $C_4$, does not involve any death, division or differentiation events ($\mu _4=\lambda _4=\xi _4=0$), to represent the terminal accumulation of cells in it. This allows us to quantify the number of cells that *exit* the system formed by the first three compartments, which is of interest in processes such as thymic development^14^. We choose $\mu _i=1=\mu$ for all $i\in \{1,2,3\}$, so that the unit of time for the system is the mean lifetime of a cell. We want to study the impact of asymmetric and symmetric division on the dynamics, and thus, choose $\nu _1=\nu _2=\nu _3=1/2$ in the irreversible model, so that all compartments have the same differentiation rates.

Cells can divide in each compartment with per-cell rate $\omega$, and this division represents self-renewal with probability $p_{SR}$, asymmetric division with probability $p_{AD}$, and symmetric division with probability $p_{SD}$. This is equivalent to setting, with the notation introduced in Section “Stochastic compartmental model”, $\lambda _i= p_{SR}\omega$, $s_i=p_{SD}\omega$ and $a_i=p_{AD}\omega$, for $i\in \{1,2,3\}$. We choose $\omega =0.9<1.0=\mu$, so that the system has significant proliferative potential, and focus on the following scenarios:

*Only-SR.*: $$p_{SR}=1.0$$, $$p_{SD}=0$$, $$p_{AD}=0$$.
*Dominant-SR.*: $$p_{SR}=0.8$$, $$p_{SD}=0.1$$, $$p_{AD}=0.1$$.
*Dominant-SD.*: $$p_{SR}=0.1$$, $$p_{SD}=0.8$$, $$p_{AD}=0.1$$.
*Dominant-AD.*: $$p_{SR}=0.1$$, $$p_{SD}=0.1$$, $$p_{AD}=0.8$$.

**Figure 5:**
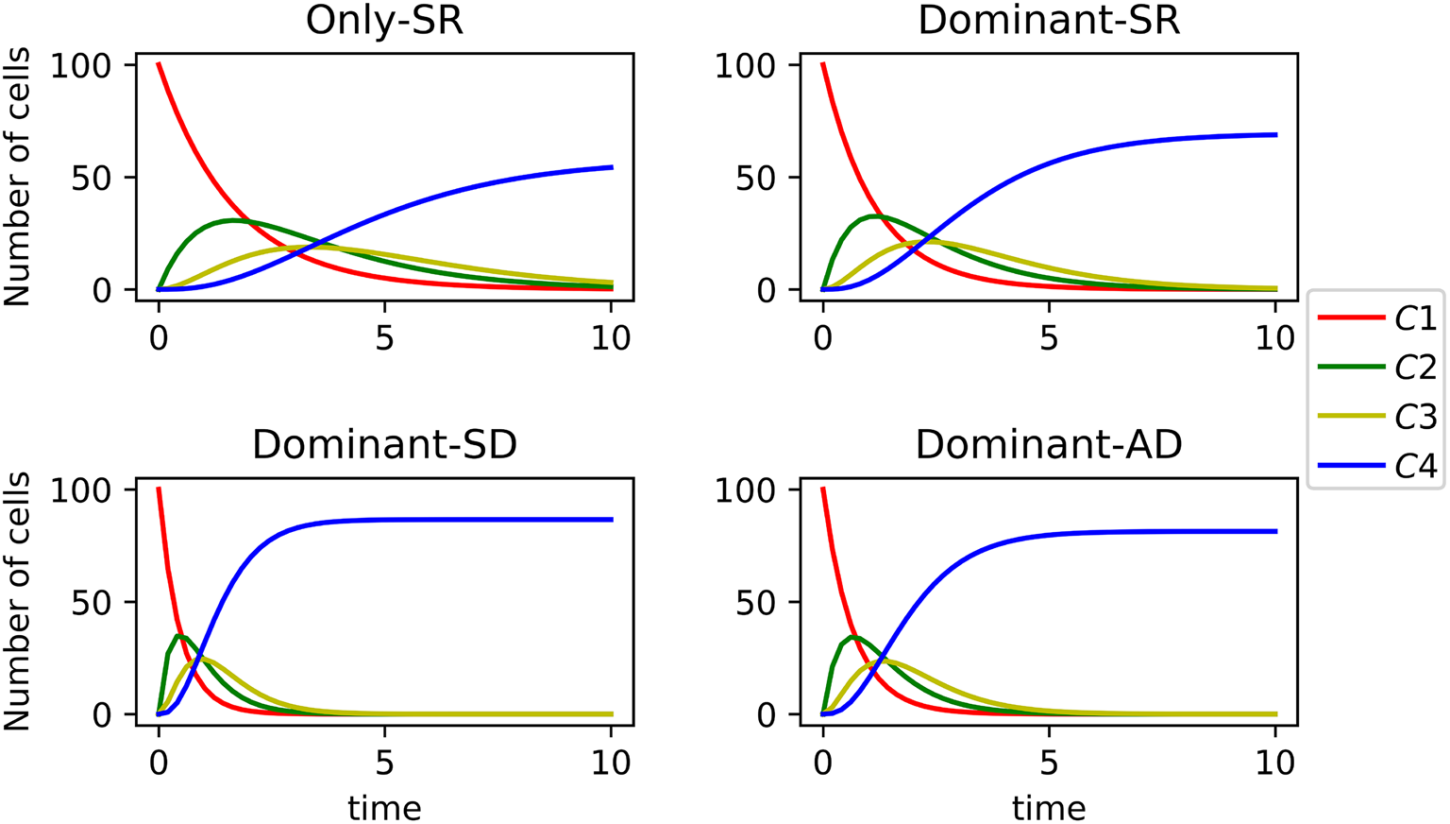
Dynamics of the mean number of cells, ${\mathbb {E}}[{\varvec{C}}_i(t)]$, in compartments $i\in \{1,2,3,4\}$ for each of the four scenarios considered in Section “Asymmetric and symmetric division: the case of four compartments”.

Our aim in this section is to explore the impact that asymmetric or symmetric division has on the dynamics of the system (dominant-SR/SD/AD scenarios), compared to the situation where only self-renewal takes place (only-SR scenario). In Fig. 5 we plot the mean number of cells, ${\mathbb {E}}[{\varvec{C}}_i(t)]$, in compartments $i\in \{1,2,3,4\}$ for each scenario. For compartments $C_i$ with $i\in \{1,2,3\}$, and since $\Delta =\Delta _1=\Delta _2=\Delta _3$ and $\Lambda =\Lambda _1=\Lambda _2=\Lambda _3$, one can directly make use of Eq. (6). For compartment $C_N$, $N=4$, one has

$$\begin{aligned} {\mathbb {E}}[{\varvec{C}}_{N}(t)]= & {} \left( \frac{\Lambda }{\Delta } \right) ^{N-1}-\sum _{j=1}^{N-1} {\mathbb {E}}[{\varvec{C}}_j(t)] \left( \frac{\Lambda }{\Delta } \right) ^{N-j}. \end{aligned}$$

In Fig. 5, we consider initial conditions ${\varvec{C}}_1(0)=10^2$, ${\varvec{C}}_2(0)={\varvec{C}}_3(0)={\varvec{C}}_4(0)=0$, representing $10^2$ initial cells in the first compartment and no cells in the other ones. We observe that an exponential decay in the number of cells in $C_1$ is followed by sequential increases in the subsequent compartments, until a steady-state number of cells is achieved in the *terminal* compartment, $C_4$. Interestingly, the dynamics is faster if symmetric or asymmetric division is considered (dominant-SR/SD/AD scenarios compared to only-SR): the decay in compartment $C_1$ is quicker and the steady-state is reached sooner. The fastest dynamics is observed for the dominant-SD case, where symmetric division is more likely, and the two daughters of a cell move directly to the next compartment. Figure 5 indicates that symmetric or asymmetric division does not only affect the dynamics but also the total mean cell number exiting the system (i.e., reaching the terminal compartment, $C_4$). This is directly related to the fact that cells can die at any stage in the differentiation process. In particular, $\lim _{t\rightarrow +\infty }{\mathbb {E}}[{\varvec{C}}_4(t)]$ is significantly larger when asymmetric and (especially) symmetric division can occur. We note that, importantly, the division rate, $\omega$, is equal in all four scenarios. This suggests that, in this type of systems, asymmetric or symmetric division (compared to self-renewal division) facilitates the generation of a larger terminally differentiated population with the same overall *proliferative capacity*; that is, in the only-SR scenario, a larger number of divisions would be required in each compartment for enough cells to escape death and differentiate to the next compartment, and to eventually reach $C_4$.

**Figure 6:**
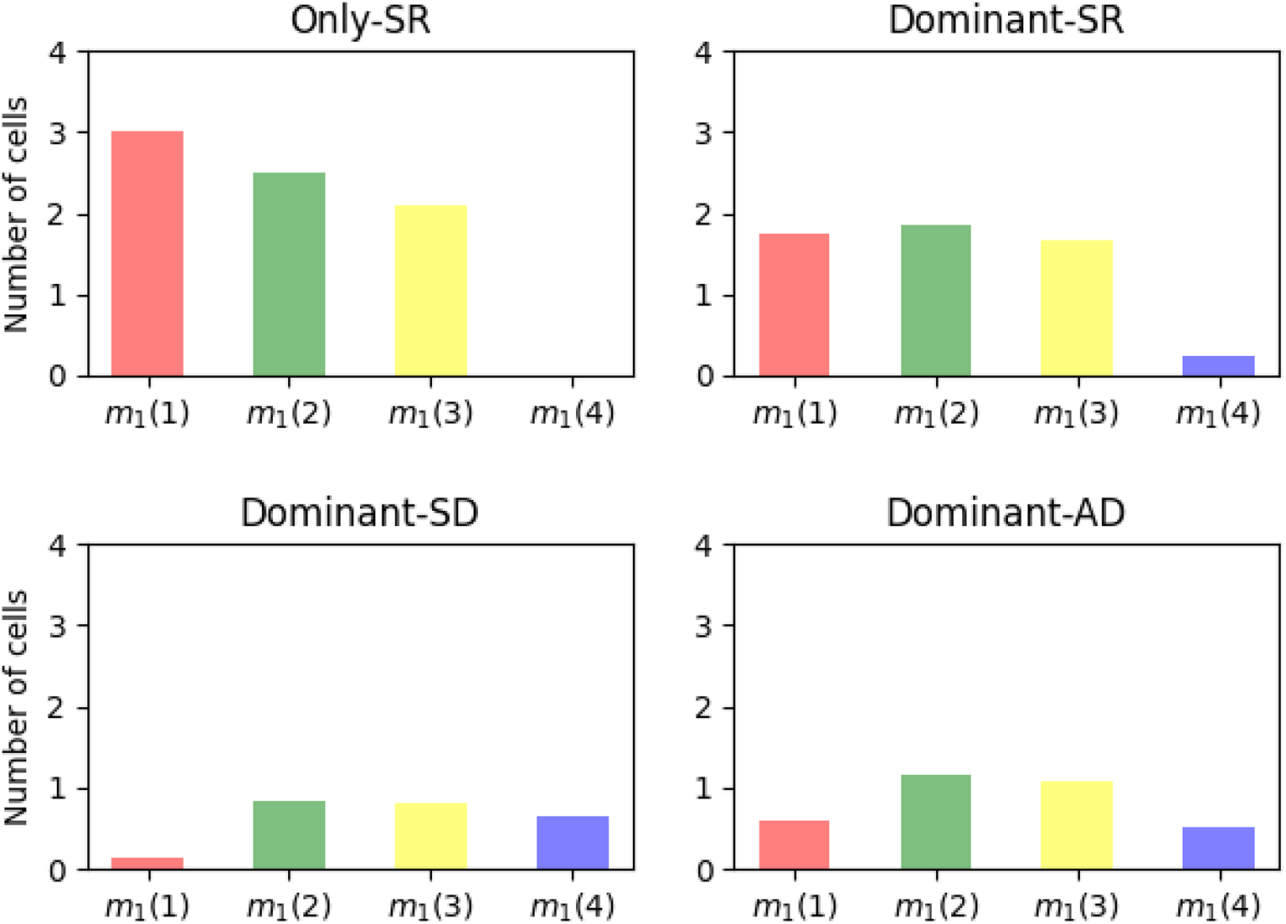
Mean number of cells $m_1(j)$ in the progeny of a single cell starting in $C_1$, belonging to compartments $C_j$, $j\in \{1,2,3,4\}$, for all four scenarios.

Our comments above are consistent with the results shown in Fig. 6, where we plot the mean number of cells, $m_1(j)$, in the progeny of a single cell starting in $C_1$, belonging to compartments $C_j$, $j\in \{1,2,3,4\}$, for all four scenarios. In the only-SR scenario, the mean number of cells in the progeny of the progenitor cell decreases monotonically across the sequence of compartments, $m_1(1)>m_1(2)>m_1(3)>m_1(4)$. We note that $m_1(4)=0$ can be explained since it only accounts for progeny cells which arrive into compartment $C_4$ as a direct result of cell proliferation, and no symmetric or asymmetric division is considered in the only-SR scenario. We also stress here that this monotonic decrease happens even though the division and differentiation rates are equal in all compartments $j\in \{1,2,3\}$. This can be explained by the fact that some cells in the progeny will die before reaching compartments $C_2$ or $C_3$. On the other hand, and as discussed in Fig. 5, scenario only-SR leads to the largest mean progeny, $m_1=m_1(1)+m_1(2)+m_1(3)+m_1(4)$. This suggests that the only-SR scenario is an *inefficient* way to reach a desired population size of terminal cells. Asymmetric and (especially) symmetric division events significantly reduce the number of descendants from a single progenitor cell in all compartments, while maximising the number of terminal (or product) cells (see Fig. 5). In particular, the dominant-SD scenario is characterised by the highest total mean number of terminal cells, ${\mathbb {E}}[{\varvec{C}}_4(+\infty )]$, as well as the smallest progeny size, $m_1$, while leading to the largest progeny, $m_1(4)$, in the terminal compartment.

Finally, the fastest dynamics observed in scenarios with symmetric division, as well as the reduced progeny observed from a single progenitor in $C_1$, imply that this kind of systems can go from unbounded growth to population extinction at late times, by increasing the number of symmetric division events. We explore this further in the next section, looking at a particular case study.

### Hematopoietic stem cells: self-renewal, asymmetric and symmetric division

We consider the model proposed in Ref.[^9^, Figure 6A] for the gradual differentiation of hematopoietic stem cells (HSCs). HSCs are responsible for the production of all blood cells^52^. In order to maintain such an heterogeneous population, HSCs are able to self-renew and differentiate. Moreover, HSCs need to act continuously and rapidly to either replace short-lived blood cells, or respond to hematopoietic stress arising from events such as bleeding and toxin spotting^53^. Recent advances in flow cytometry and single-cell analysis have shown that HSC cells are a small population compared to the many other blood cell types they can generate. Despite their importance, the molecular mechanisms involved in hematopoietic stem cell maintenance remain unclear.

In order to study simultaneously HSC proliferation and differentiation, Barile et al.^9^ propose a novel mathematical model inferred by cell-cycle dependent labelling and HCS fate mapping data. Cells at each state can undergo five different processes: self-renewal, asymmetric cell division, symmetric cell division, direct differentiation and cell death. This leads to the following compartmental sequence: HSC1 $\rightarrow$ HSC2 $\rightarrow$ MPP1+MPP2 $\rightarrow$ MPP3 $\rightarrow$ HPC1 (see Ref.[^9^, Figure 6A]) consisting of $N=5$ different compartments. These represent two stages of hematopoietic stem cells (HSC1 and HSC2), two stages of multi-potent progenitor cells (MPP1 + MPP2 and MPP3), and a final stage of hematopoietic progenitor cells (HPC1).

**Table 1:** Parameter values obtained from Ref.[^9^, Figure 6], where they set $a_i=s_i=0$ for all $i\in \{1,\ldots ,5\}$.

	$\Delta _i$	$\Lambda _i$
HSC1	− 0.0046197	0.016497
HSC2	0.0017357	0.007847
MPP1+2	0.0044844	0.032834
MPP3	0.01556	0.16113
HPC1	0.0293	0

All parameters reported in this section have units of days^−1^.

**Figure 7:**
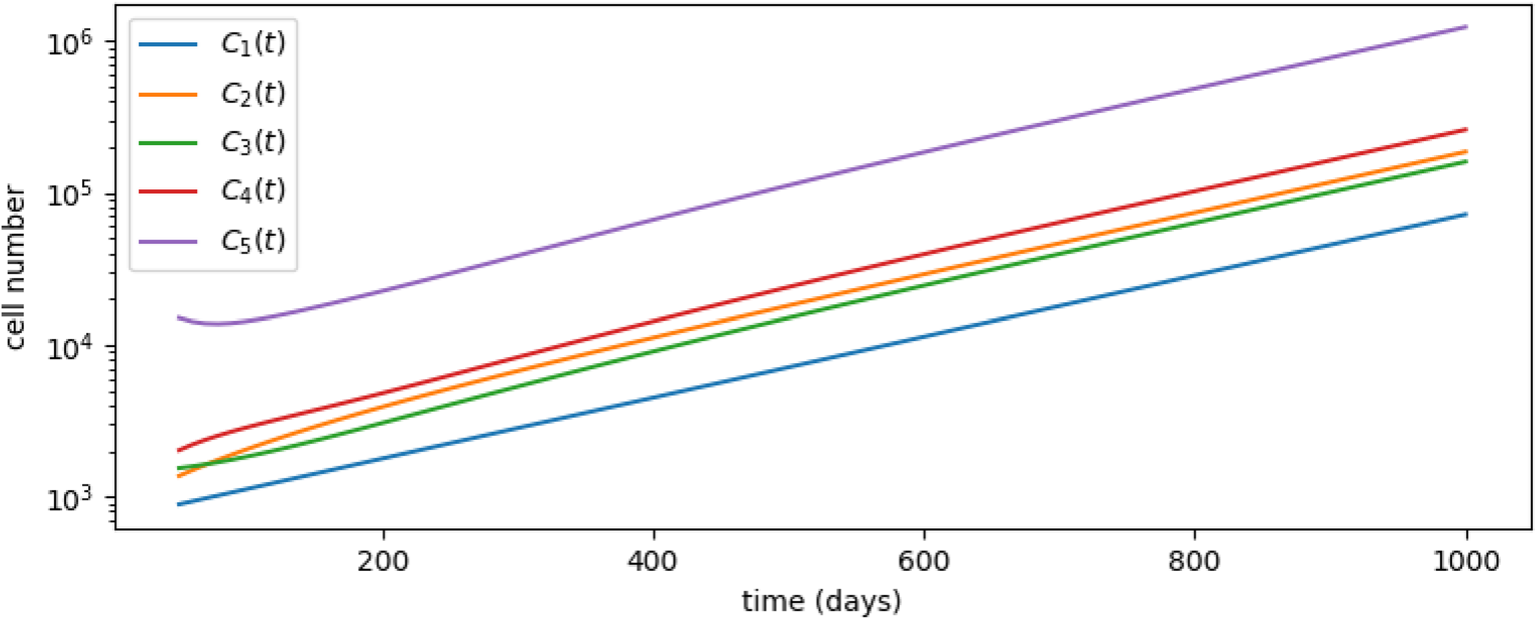
Dynamics of the ODEs system (1) and parameters from Table 1, corresponding to the only-SR scenario. We choose initial conditions $(C_1(0),C_2(0),C_3(0),C_4(0),C_5(0))=(890, 1370, 1540, 2020, 1.5\times 10^4)$, taken from Ref.[^9^, Figure S1 H].

The model proposed by Barile et al.^9^ corresponds to the irreversible model (i.e., $\xi _i=0$ for $i\in \{2,\ldots ,5\}$) shown in Fig. 2, when we set $N=5$ compartments and consider the parameter values in Table 1, from Ref.[^9^, Figure 6]. We note that the “net loss rate” for HSC1 cells, $\Delta _1=-4.6197\times 10^{-3}$ days^−1^, is negative. Thus, the growth of the HSC1 population is unbounded, as can be observed in Ref.[^9^, Figure S1 H]. This is also shown in Fig. 7, where we simulate the system given by Eq. (2) for the parameters of Table 1.

We note that the parameter calibration performed by Barile et al.^9^ predicted relatively negligible symmetric and asymmetric division rates for most of the compartments. Thus, these authors assumed $s_i=a_i=0$ for all $i\in \{1,\ldots ,5\}$, as given in Table 1 and Fig. 7. We now explore, for this system, the potential role that symmetric and asymmetric division could play. To that end, we perform a sensitivity analysis on the parameters $(s_i,a_i)$, for $i\in \{1,\ldots ,5\}$. In particular, we consider four different scenarios:

- *Only-SR.* All parameters as in Table 1, where $s_i=a_i=0$ for $i\in \{1,\ldots ,5\}$ and division events correspond to self-renewal, as reported by Barile et al.^9^, and shown in Fig. 7.
- *Symm1.* Symmetric division rate $s_1\in \{10^{-2},10^{-1}\}$ for compartment HSC1 is added to the rates in Table 1, with $s_i=0$ for $i\in \{2,\ldots ,5\}$, and $a_i=0$ for $i\in \{1,\ldots ,5\}$.
- *SymmAll.* Equal symmetric division rates $s_i \in \{10^{-2},10^{-1}\}$ per day, for $i\in \{1,\ldots ,5\}$ are added to the rates in Table 1, with $a_i=0$ for $i\in \{1,\ldots ,5\}$.
- *AsymmAll.* Equal asymmetric division rates $a_i \in \{10^{-4},10^{-3},10^{-2},10^{-1}\}$ per day, $i\in \{1,\ldots ,5\}$ are added to the rates in Table 1. Symmetric division only occurs in compartment HSC1, $s_1=5\times 10^{-3} {day^{-1}}$, chosen so that population extinction is guaranteed.

**Figure 8:**
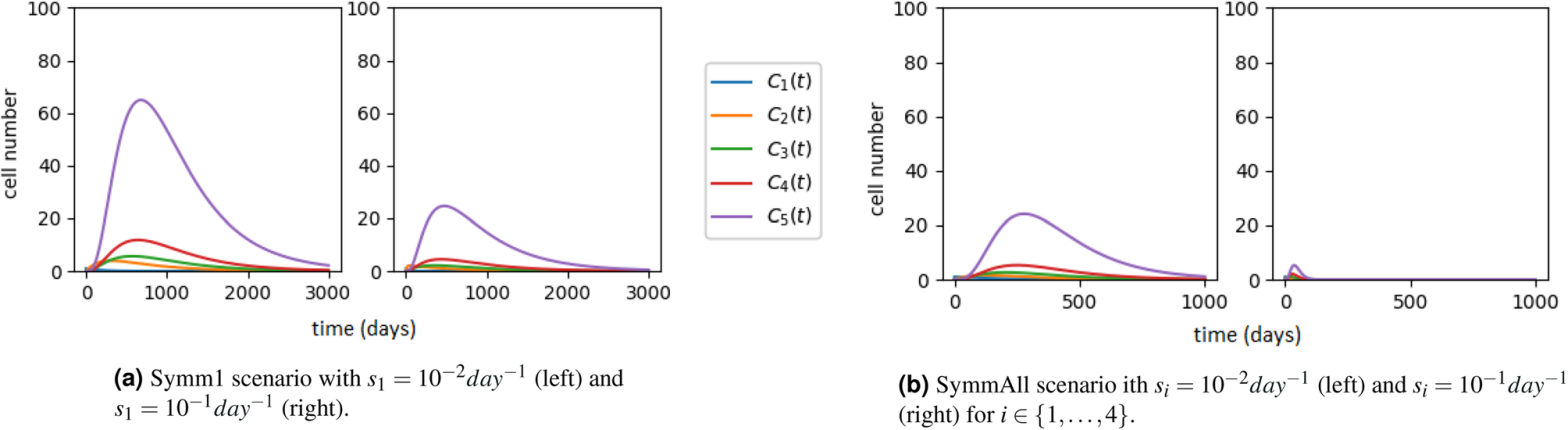
Dynamics of the ODE system (1) with initial conditions $(C_1(0),C_2(0),C_3(0),C_4(0),C_5(0)) = (1,0,0,0,0)$. Parameter values as in Table 1, except for symmetric division rates. (**a**) Scenario Symm1, where the symmetric division rate $s_1$ is positive only in the HSC1 compartment. (**b**) Scenario SymmAll, where the symmetric division rate $s_i$ is positive and identical for all compartments.

The net loss rate for compartment *i*, $\Delta _i\equiv \mu _i+\nu _i+s_i-\lambda _i$, does not depend on the asymmetric division rate, $a_i$, but it does depend on the symmetric rate, $s_i$. Thus, just by tuning the symmetric division rate, $s_1$, of HSC1 cells (Symm1 scenario), we can drastically change the dynamics of the entire system: from unbounded growth (Fig. 7) to population extinction (Fig. 8a). Furthermore, as shown in Fig. 8b, if all the compartments are characterised by a non-zero symmetric division rate (SymmAll scenario), then the resulting population size is smaller but the overall dynamics faster. The change in the asymptotic behaviour of the system, from unbounded growth to extinction, and the smaller number of HSC cells observed in the SymmAll scenario compared to the Symm1 one, can be explained by the transitions across compartments generated by symmetric division events. When a symmetric division event occurs in compartment $C_i$, two cells in the subsequent compartment $C_{i+1}$ are generated, while the number of cells in compartment $C_i$ decreases by one. Thus, symmetric division events speed up the transition of cells to subsequent compartments and, at the same time, deplete cells from the compartment where the division took place.

**Figure 9:**
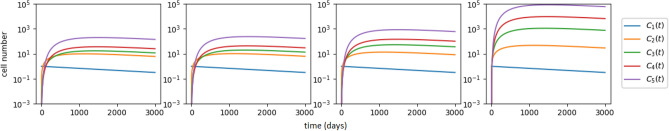
Dynamics of the system (1) and parameter values as in Table 1, except for $s_1=5\times 10^{-3}$ day^−1^ and asymmetric division rates. AsymmAll scenario with equal asymmetric division rates $a_i\in \{10^{-4}, 10^{-3}, 10^{-2}, 10^{-1}\}$ per day, in all compartments, and initial conditions $(C_1(0),C_2(0),C_3(0),C_4(0),C_5(0)) = (1,0,0,0,0)$.

In Fig. 9 we explore the impact of increasing the asymmetric division rates, by considering the AsymmAll scenario, where those rates are positive and identical for all compartments. We note that, since asymmetric division rates do not affect the asymptotic qualitative behaviour of the system (i.e., unbounded growth versus extinction), we set $s_1=5\times 10^{-3}{day^{-1}}$, so that population extinction is guaranteed. When comparing the AsymmAll, Symm1 and SymmAll scenarios, it is clear that increasing the asymmetric division rates leads to a greater number of cells across compartments. This is due to the fact that asymmetric division events increase the number of cells in subsequent compartments without depleting the compartment where the division takes place. In practice, for situations where population extinction is guaranteed at late times (Fig. 9), increasing the asymmetric division rates delays the time when extinction occurs. We note Fig. 9 differs from Fig. 6, but they are not in contradiction. In Fig. 6, the division rate $\omega$ is kept constant, while the probability for each type of division event is not, but given by $(p_{SR},p_{SD},p_{AD})$. In Fig. 9, we increase the asymmetric division rate in each compartment instead, effectively increasing the division rate $\omega$, and thus, leading to a greater population size over time in the system.

**Figure 10:**
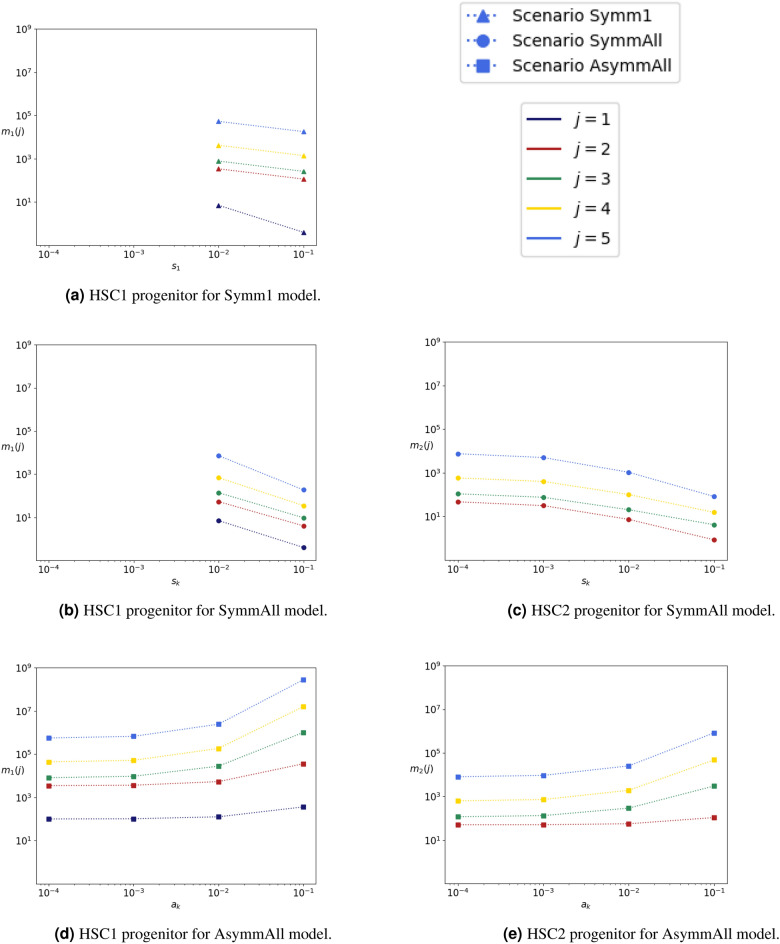
Mean number, $m_i(j)$, of cells in the progeny for an (**a**, **b**, **d**) HSC1 ($i=1$) or (**c**, **e**) HSC2 ($i=2$) progenitor, in compartments $j\in \{i,\ldots ,5\}$. In each scenario, we vary the corresponding rate ($s_1$ in Symm1, $s_k$, $k\in \{1,\ldots ,N\}$ in SymmAll, and $a_k$, $k\in \{1,\ldots ,N\}$ in AsymmAll), where only values leading to finite $m_i(j)$ are considered.

Studies suggest that very low numbers of HSC cells (HSC1 and HSC2) can maintain a continuous stream of differentiating cells and generate a large number of mature blood cells^8,52^. During hematopoiesis, HSCs cells slowly replace short-lived MPP cells. This heterogeneous population no longer possesses self-renewal ability but still retains differentiation potential^52^. We note here that the parameter values estimated by Barile et al.^9^ result in an almost zero net loss rate for HSC2s and MPP1+MPP2 cells; that is, $\Delta _2 \approx \Delta _3 \approx 0$. This agrees with the hypothesis that the self-renewal rate of HSC2 and MPP1+MPP2 cells is sufficient to maintain, alone, the populations of more differentiated cells (e.g., MPP3, HPC1), with minimal input from HSC1 cells. Thus, it is pertinent to study the progeny of a single HSC (HSC1 or HSC2), and study how symmetric and asymmetric division events influence it. To do this, we implement Eq. (14) in the irreversible model for $i\in \{1,2\}$ and $j\in \{i,\ldots ,5\}$, and compute $m_i(j)$, the mean number of cells within the progeny of a single cell from compartment $C_i$ (HSC1 or HSC2) in subsequent compartments $C_j$, $j\in \{i,\ldots ,5\}$. The results are shown in Fig. 10, where we plot $m_i(j)$ for the Symm1, SymmAll and AsymmAll scenarios, for $i\in \{1,2\}$ and $j\in \{i,\ldots ,5\}$. We note that the Symm1 scenario is not considered in Fig. 10 for $i=2$, since changes in the symmetric division rate, $s_1$, do not affect $m_2(j)$. First, we observe that the mean number of cells in the progeny of a single HSC1 progenitor across compartments, $m_1(j)$, increases for increasing values of *j*; that is, for more differentiated cells regardless of the scenario. Indeed, most cells within the progeny of a single HSC1 progenitor belong to the last HPC compartment, consistent with the dynamics observed in Figs. 8 and 9.

The trend of $m_1(j)$ is drastically different for scenario AsymmAll compared to scenarios Symm1 or SymmAll, for increasing values of the corresponding rate ($s_1$ in Symm1, $s_k$, $k\in \{1,\ldots ,N\}$ in SymmAll, and $a_k$, $k\in \{1,\ldots ,N\}$ in AsymmAll). In the AsymmAll scenario, $m_1(j)$ is an increasing function of the asymmetric division rate $a_k$, whereas in the Symm1 and SymmAll scenarios, $m_1(j)$ is a decreasing function of the symmetric division rate. This agrees with the dynamics shown in Fig. 8, where increasing values of the symmetric division rate prevent population growth, guaranteeing extinction at late times. An increase of the asymmetric division rate leads to significant production of MPP and HPC cells (see $m_1(4)$ and $m_1(5)$ in Fig. 10) within the progeny, and thus, it could potentially play a role in situations of hematopoietic stress. Similar behaviour can be observed for $m_2(j)$. In agreement with $m_1(j)$ in Fig. 10, we observe a decrease in $m_2(j)$ for scenario SymmAll and an increase in $m_2(j)$ for scenario AsymmAll, as a function of the corresponding division rate. Finally, we compare the Symm1 and SymmAll scenarios. We observe that when the symmetric division rate equals $10^{-2}$ per day, the number of cells in the progeny of a single HSC1 progenitor differs by almost an order of magnitude between the Symm1 and SymmAll scenarios; that is, symmetric division events taking place in all compartments (SymmAll) lead to smaller progeny from an initial HSC1 cell in subsequent compartments, compared to the Symm1 scenario. When increasing the symmetric division rate to $10^{-1}$ per day, the difference between the number of cells, $m_1(j)$, within the progeny for the Symm1 and SymmAll scenarios increases further. Clonal hematopoiesis that has been observed in older mice and humans^54^ is strictly linked to the accumulation of mutations in the HPC population and an increasing risk of leukemia. Thus, our results suggest that symmetric division could be a possible way to control cell differentiation and limit mutation accumulation in the hematopoietic system.

### Tracking a thymocyte during its development

**Figure 11:**
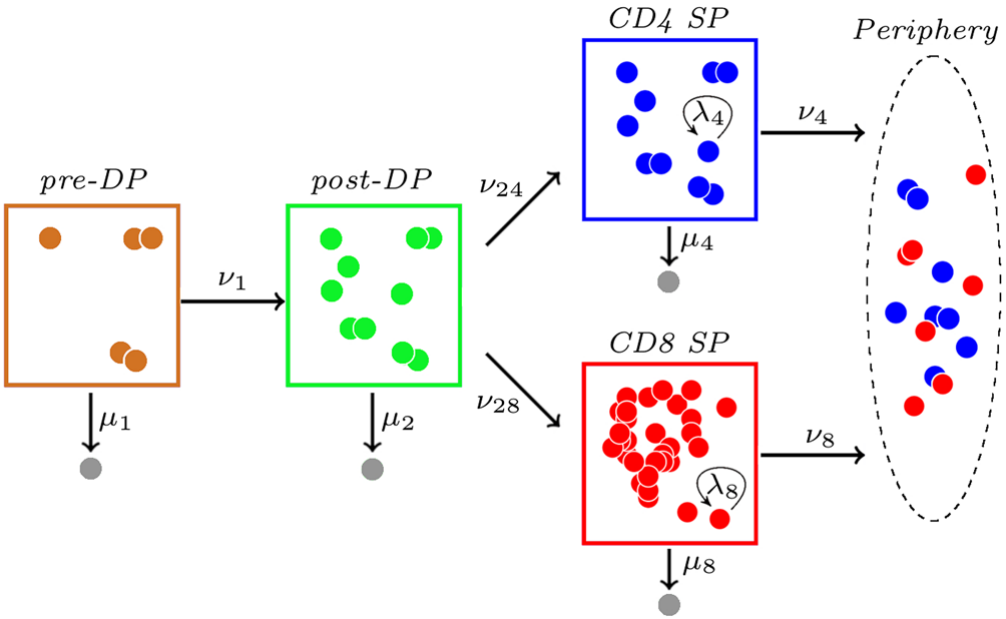
Thymic development model proposed in Ref.^14^. Grey cells represent death events.

We now consider the T cell thymic development model proposed in Ref.[^14^, Model 2], and shown in Fig. 11. Double negative (DN) thymocytes differentiate to become pre-selection DP thymocytes (pre-DP). In this model, pre-DP is the first compartment, which will contain an initial number of cells (initial condition, $C_1(0)$). Pre-DPs undergo maturation in the thymus. These cells can progress to the double positive stage (post-DP), where thymocytes express both CD4 and CD8 co-receptors. Post-DP cells that are positively selected transition to the single positive (SP) stage, where they can express either the CD4 or CD8 co-receptor. Some of these cells will then reach the periphery as (single) CD4 or CD8 SP cells.

We exploit this particular model to illustrate the applicability of the analysis developed in Section “Lifeline analysis”. This case study also allows us to show how these methods can easily be adapted to different compartment topologies. In this case, we have a compartmental bifurcation, rather than a linear sequence of compartments.

First, it is of interest to estimate the percentage of pre-DP thymocytes that are predicted to die in each of the compartments during development, and the percentage that successfully reach the periphery instead (either as a CD4 or CD8 SP cell). It is clear that our arguments in Section “Lifeline analysis” can easily be adapted to do so. In particular, one can slightly redefine the probabilities $\beta _i(j)$ in Section “Lifeline analysis”, with $i=1$ (i.e., a single pre-DP thymocyte being tracked), as

$$\begin{aligned} \beta _{1}(1)= & {} \hbox {probability\;that\;the\;pre-DP\;thymocyte\;dies\;in\;the\;pre-DP\;compartment},\\ \beta _{1}(2)= & {} \hbox {probability\;that\;the\;pre-DP\;thymocyte\;dies\;in\;the\;post-DP\;compartment},\\ \beta _{1}(4)= & {} \hbox {probability\;that\;the\;pre-DP\;thymocyte\;dies\;in\;the\;CD4 SP\; compartment},\\ \beta _{1}(8)= & {} \hbox {probability that the pre-DP thymocyte dies in the CD8 SP compartment},\\ \beta _{1}(4P)= & {} \hbox {probability that the pre-DP thymocyte reaches the periphery as a CD4 SP cell}, \\ \beta _{1}(8P)= & {} \hbox {probability that the pre-DP thymocyte reaches the periphery as a CD8 SP cell}. \end{aligned}$$

Similar solutions to those derived in Section “Lifeline analysis” can be obtained by incorporating the compartmental bifurcation in the first-step analysis, leading to

$$\begin{aligned} \beta _{1}(1)= & {} \frac{\mu _1}{\mu _1+\nu _1},\\ \beta _{1}(2)= & {} \frac{\nu _1}{\mu _1+\nu _1}\frac{\mu _2}{\mu _2+\nu _{24}+\nu _{28}},\\ \beta _{1}(4)= & {} \frac{\nu _1}{\mu _1+\nu _1}\frac{\nu _{24}}{\mu _2+\nu _{24}+\nu _{28}}\frac{\mu _4}{\mu _4+\nu _4},\\ \beta _{1}(8)= & {} \frac{\nu _1}{\mu _1+\nu _1}\frac{\nu _{28}}{\mu _2+\nu _{24}+\nu _{28}}\frac{\mu _8}{\mu _8+\nu _8},\\ \beta _{1}(4P)= & {} \frac{\nu _1}{\mu _1+\nu _1}\frac{\nu _{24}}{\mu _2+\nu _{24}+\nu _{28}}\frac{\nu _4}{\mu _4+\nu _4},\\ \beta _{1}(8P)= & {} \frac{\nu _1}{\mu _1+\nu _1}\frac{\nu _{28}}{\mu _2+\nu _{24}+\nu _{28}}\frac{\nu _8}{\mu _8+\nu _8}. \end{aligned}$$

These analytical expressions allow us to perform a local sensitivity analysis by computing partial derivatives with respect to model parameters. For example, we have

$$\begin{aligned} \frac{\partial \beta _1(8P)}{\partial \nu _{24}}= & {} \frac{\partial \beta _1(8P)}{\partial \mu _{2}} \ = \ - \frac{\nu _1\,\nu _{28}\,\mu _8}{(\mu _1+\nu _1)(\mu _2+\nu _{24}+\nu _{28})^2(\mu _8+\nu _8)}. \end{aligned}$$

The proliferative potential of thymocytes during thymic development directly depends on them reaching the CD4 SP or CD8 SP compartment, where they are able to divide, before they exit to the periphery. Thus, the average number of divisions initiated by a single pre-DP thymocyte during its thymic development journey, $\eta _1=\eta _1(4)+\eta _1(8)$, is given by

$$\begin{aligned} \eta _1(4)= & {} \frac{\nu _1\nu _{24}}{(\mu _1+\nu _1)(\mu _2+\nu _{24}+\nu _{28})}\frac{\lambda _4}{\mu _4+\nu _4},\\ \eta _1(8)= & {} \frac{\nu _1\nu _{28}}{(\mu _1+\nu _1)(\mu _2+\nu _{24}+\nu _{28})}\frac{\lambda _8}{\mu _8+\nu _8}. \end{aligned}$$

Finally, the average lifespan of a pre-DP cell during thymic development (i.e., the mean time until it dies or it reaches the periphery) is given by

$$\begin{aligned} \tau _1 = \frac{1}{\mu _1+\nu _1} \left[ \frac{\nu _1}{\mu _2+\nu _{24}+\nu _{28}}\left( \frac{\nu _{24}}{\mu _4+\nu _4}+\frac{\nu _{28}}{\mu _8+\nu _8}+1\right) +1\right] . \end{aligned}$$

**Table 2:** Parameter values from Ref.[^14^, Section 3.2], in units $days^{-1}$.

Rate	$\mu _1$	$\nu _1$	$\mu _2$	$\nu _{24}$	$\nu _{28}$	$\lambda _4$	$\lambda _8$	$\mu _4$	$\mu _8$	$\nu _4$	$\nu _8$
Value	0.263	0.137	1.369	0.07	0.054	0.216	0.093	0.04	0.11	0.21	0.14

**Figure 12:**
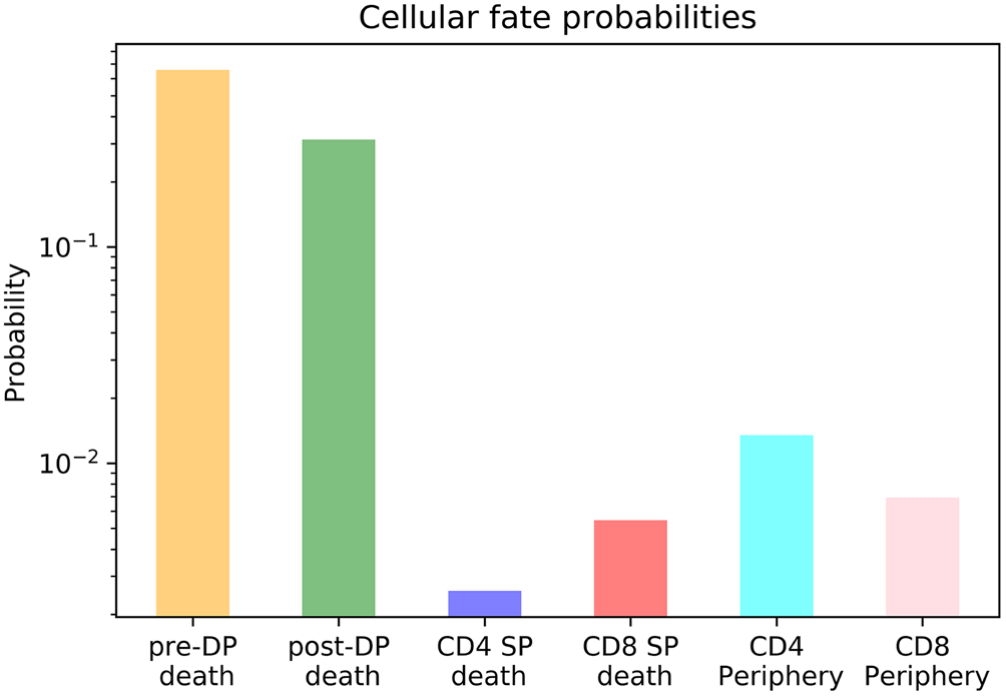
Probabilities of a single pre-DP cell to die in each of the compartments (pre-DP, post-DP, CD4 SP, or CD8 SP) before reaching the periphery, or to reach the periphery as a CD4 or CD8 SP cell. In particular, $\beta _1(1)=0.6575$, $\beta _1(2)=0.3140$, $\beta _1(4)=0.0026$, $\beta _1(8)=0.0055$, $\beta _1(4P)= 0.0135$ and $\beta _1(8P)=0.0069$.

We consider parameter values in Table 2 selected from Ref.[^14^, Section 3.2], to compute the average lifespan of a pre-DP cell during its thymic development journey (until it dies or reaches the periphery), which corresponds to $\tau _1=2.84$ days. During its lifetime, a cell may undergo differentiation and proliferation, before dying in one of the compartments without ever reaching the periphery, or reaching the periphery either as a CD4 or CD8 cell. We show the predicted cell fate probabilities in Fig. 12. The most likely outcome corresponds to cell death, especially during the early stages (pre-DP and post-DP compartments). This agrees with existing evidence that most of negative selection occurs during the DP stages of development^55^. Once a cell reaches the CD4 SP compartment, it is more likely to reach the periphery than to die in that compartment, while these probabilities are comparable in the CD8 SP case.

In Table 3, we present the elasticities (i.e., normalised derivatives) of the probabilities $\beta _1(j)$, $j\in \{1,2,4,8,4P,8P\}$, with respect to model parameters. This can be of particular relevance when parameters have been experimentally estimated with some uncertainty, so that one can assess the impact of perturbations in these values on specific model outputs. As expected, the division rates, $\lambda _4, \lambda _8$, do not affect the probability of death of the tracked cell starting as pre-DP. Moreover, the death rates $\mu _1,\mu _2,\mu _4,\mu _8$ positively contribute to the probability of the cell dying in the corresponding compartment $j\in \{1,2,4,8\}$, while negatively contributing to the probability of the cell dying in other compartments. It is also worth noting that, for $j\in \{2,4,8\}$, the probability, $\beta _1(j)$, of the cell dying in that compartment is mainly affected by the differentiation rate into compartment *j*; that is, $\nu _1,\nu _{24},\nu _{28}$, respectively. This can be understood since we are tracking a cell starting in compartment $i=1$, and following its developmental journey across the sequence of compartments. Thus, the probability of dying in a compartment $j\ne 1$ is mainly determined by the differentiation rates of previous compartments.

**Table 3:** Elasticities for the probabilities $\beta _1(j)$, $j\in \{1,2,3,8,4P,8P\}$, with respect to parameter $\theta \in \{ \mu _1,\nu _1,\mu _2,\nu _{24},\nu _{28}, \lambda _4,\lambda _8,\mu _4,\mu _8,\nu _4,\nu _8\}$. They are given by $(\partial \beta _1(j)/ \partial \theta )/(\beta _i(j)/ \theta )$, with cell fate probabilities (in rows) and model parameters (in columns).

$\frac{\partial \beta _i(j)}{\partial \theta }/\frac{\beta _i(j)}{\theta }$	$\mu _1$	$\nu _1$	$\mu _2$	$\nu _{24}$	$\nu _{28}$	$\lambda _4$	$\lambda _8$	$\mu _4$	$\mu _8$	$\nu _4$	$\nu _8$
$\beta _1(1)$	0.34	$-0.34$	0	0	0	0	0	0	0	0	0
$\beta _1(2)$	$-0.66$	0.66	0.08	$-0.04$	$-0.04$	0	0	0	0	0	0
$\beta _1(4)$	$-0.66$	0.66	$-0.92$	0.96	$-0.04$	0	0	0.84	0	$-0.84$	0
$\beta _1(8)$	$-0.66$	0.66	$-0.92$	$-0.04$	0.96	0	0	0	0.56	0	$-0.56$
$\beta _1(4P)$	$-0.66$	0.66	$-0.92$	0.96	$-0.04$	0	0	$-0.16$	0	0.16	0
$\beta _1(8P)$	$-0.66$	0.66	$-0.92$	$-0.04$	0.96	0	0	0	$-0.44$	0	0.44

The average number of division events performed by a single pre-DP cell is $\eta _1=\eta _1(4)+\eta _1(8)=0.0139+0.0046=0.0185$. This implies that out of $10^2$ pre-DP cells starting the thymic development journey only (about) 2 cells are expected to be produced by cell division from the original cells, when visiting the CD4 SP or CD8 SP compartments. These small values are directly related to the small probabilities of reaching these compartments at all, so that the cell can actually divide. Our results here are in agreement with the results from Ref.^14^, where the authors make use of a deterministic model to conclude that thymic development is a rather stringent process characterised by an extremely low success rate.

## Discussion

We have presented a general model to characterise the stochastic journeys of cell progenies through compartments. Cells can divide, die or exit to adjacent compartments. We have derived analytical expressions for the mean number of cells in each compartment as a function of time, under different scenarios of interest (e.g., irreversible model, where differentiation cannot be reversed) and studied the progeny of a single progenitor cell in terms of the probability generating function and summary statistics appropriately defined. The analysis allows us to track the journey of a lifeline across the system of compartments. We have then calculated its lifetime, its proliferative potential and the probability of different cell fates. We have used case studies to illustrate the applicability of our techniques and the impact of model parameters on the corresponding summary statistics.

The analysis carried out in Section “Lifeline analysis” sheds light on the dynamics of the cell population by analysing the journey followed by a single cell. Moreover, this technique is rather “flexible” since the first-step arguments in which it relies can be easily extended to other compartmental topologies, as we have briefly illustrated in the third case study (see Section “Tracking a thymocyte during its development”). Moreover, novel labelling and barcoding techniques provide an increasing amount of data^56–59^, which could be compared to this type of model predictions.

Our results also highlight the significant role that symmetric and asymmetric division events can play in these systems, when compared to self-renewal. In particular, we have shown in Section “Asymmetric and symmetric division: the case of four compartments” how symmetric division events can significantly affect the dynamics of the system, potentially moving it from unbounded growth to extinction. Increasing the asymmetric division rates does not change the late time behaviour. Still, it can delay population extinction by increasing the number of cells arising over time across compartments. On the other hand, for a fixed division rate, $\omega$, and different probabilities of each type of division event (self-renewal, $p_{SR}$; symmetric division, $p_{SD}$; asymmetric division, $p_{AD}$), compartmental systems where symmetric or asymmetric division is more likely lead to smaller cell populations and faster dynamics to extinction or steady-state, compared to systems where self-renewal is the dominant division process. Interestingly, increasing the probability of symmetric or asymmetric division leads to smaller progenies from a single progenitor cell, while maximising the size of the fully differentiated (or terminal) population. We note that, in Section “Asymmetric and symmetric division: the case of four compartments” we set all differentiation rates to be the same across compartments, since the focus was on studying the impact of symmetric/asymmetric division on the cell population dynamics. However, it is to be expected that significant heterogeneity in differentiation rates across compartments could make specific compartments more/less important in the dynamics. In particular, compartments with significantly large differentiation rates would lead to shorter residence times, which would likely imply that other events which can occur in these compartments (e.g., division) would become less relevant.

A particular limitation of our approach is that it relies on cells behaving independently from each other, as in the theory of branching processes. While this may be valid in some situations, for example when studying small cell populations where tissue growth control is only through feedback by the cell density, it might not be valid in other scenarios (e.g., for large cell populations, or under competition for resources, where logistic growth-type models might be more appropriate). This, in turn, is related to the fact that the corresponding ODE system for the average number of cells in each compartment is linear. Cell independence (or linearity) allows one to implement techniques from the theory of branching processes, and makes the single-cell analysis proposed here feasible, since we identify lifelines amongst the dynamics of all the cells in the compartmental system. Relaxing this particular assumption is, thus, the aim of future work. 

### Supplementary Information

Supplementary Information.

## Data Availability

Computer codes to generate Figures 5−10 and Figure 12 can be accessed at https://github.com/matml/Journey_Of_A_Cell_Across_A_Sequence_Of_Compartments. There is no other relevant data related to this study.
