# Supplementary material for: Stochastic journeys of cell progenies through compartments and the role of self-renewal, symmetric and asymmetric division
Source: Sci Rep. 2024 Jul 15;14:16287. doi: 10.1038/s41598-024-63500-2 (PMC11251179; doi:10.1038/s41598-024-63500-2)
Supplement: Supplementary file 1 — Supplementary Information. [file 41598_2024_63500_MOESM1_ESM.pdf]

Stochastic journeys of cell progenies through compartments and the role of self-renewal, symmetric and asymmetric division

Supplementary Material

Hanan Dreiwi[1,+], Flavia Feliciangeli[1,2,+], Mario Castro[3], Grant Lythe[1], Carmen Molina-París[4,1], and Martín López-García[1,*]

[1]School of Mathematics, University of Leeds, Leeds, UK
[2]Systems Pharmacology & Medicine, Bayer AG, Leverkusen, Germany
[3]Grupo Interdisciplinar de Sistemas Complejos (GISC), Instituto de Investigación Tecnológica (IIT), Universidad Pontificia Comillas, Madrid, Spain
[4]Theoretical Biology and Biophysics Group, Theoretical Division, Los Alamos National Laboratory, Los Alamos, NM, USA
[*]Corresponding author: m.lopezgarcia@leeds.ac.uk
[+]These authors contributed equally to this work

ABSTRACT

In this Supplementary Material, we provide Appendices A, B and C with additional details on different calculations related to Section 2 within the main manuscript.

A Mean number of cells over time in each compartment

Derivation of Equation (5)

Given the general system (1), we derive in this Appendix the solution for the irreversible model, *i.e.,* $\xi_i = 0$ for all i. The simplified system is made of linear first-order differential equations and reads

$$\begin{aligned}
\frac{d\,\mathbb{E}[\boldsymbol{C}_1(t)]}{dt} &= -\Delta_1\,\mathbb{E}[\boldsymbol{C}_1(t)] \\
\frac{d\,\mathbb{E}[\boldsymbol{C}_i(t)]}{dt} &= \Lambda_{i-1}\,\mathbb{E}[\boldsymbol{C}_{i-1}(t)] - \Delta_i\,\mathbb{E}[\boldsymbol{C}_i(t)], \quad i \in \{2,\dots,N-1\}, \\
\frac{d\,\mathbb{E}[\boldsymbol{C}_N(t)]}{dt} &= \Lambda_{N-1}\,\mathbb{E}[\boldsymbol{C}_{N-1}(t)] - \Delta_N\,\mathbb{E}[\boldsymbol{C}_N(t)],
\end{aligned} \tag{A.1}$$

where

$$\begin{aligned}
\Delta_1 &= \mu_1 + \nu_1 + s_1 - \lambda_1, \\
\Delta_i &= \mu_i + \nu_i + s_i - \lambda_i, \quad \Lambda_{i-1} = \nu_{i-1} + a_{i-1} + 2s_{i-1},\ i \in \{2,\dots,N-1\}, \\
\Delta_N &= \mu_N - \lambda_N, \quad \Lambda_{N-1} = \nu_{N-1} + a_{N-1} + 2s_{N-1}.
\end{aligned}$$

The first equation in the system (A.1) can be solved independently and the solution is

$$\mathbb{E}[\boldsymbol{C}_1(t)] = e^{-\Delta_1 t}, \tag{A.2}$$

in agreement with Eq. (5). Now, for $i = 2,3,\dots,N$ each equation of the system represents a linear first-order differential equation which requires solving the preceding equations. The general solution for each can be written as follows (see, for example, Ref.[1])

$$\mathbb{E}[\boldsymbol{C}_i(t)] = \left[\Lambda_{i-1}\int \mathbb{E}[\boldsymbol{C}_{i-1}(t)]\,e^{\Delta_i t}\,dt\ + Const_i\right] e^{-\Delta_i t},\ \ i = 2,\dots,N. \tag{A.3}$$

Following this approach, the second equation takes the form

$$\frac{d\,\mathbb{E}[\boldsymbol{C}_2(t)]}{dt} = \Lambda_1\,\mathbb{E}[\boldsymbol{C}_1(t)] - \Delta_2\,\mathbb{E}[\boldsymbol{C}_2(t)],$$

which has the following general solution

$$\begin{aligned}
\mathbb{E}[\boldsymbol{C}_2(t)] &= \left[\int \Lambda_1\,\mathbb{E}[\,\boldsymbol{C}_1(t)]\,e^{\Delta_2 t}\,dt\;+Const_2\right]\,e^{-\Delta_2 t} \\
&= \int \Lambda_1\,e^{(\Delta_2-\Delta_1)t}\,dt\,e^{-\Delta_2 t} + Const_2\,e^{-\Delta_2 t} \\
&= \Lambda_1\,\frac{e^{-\Delta_1 t}}{(\Delta_2-\Delta_1)} + Const_2\,e^{-\Delta_2 t}.
\end{aligned} \tag{A.4}$$

For the initial condition $\mathbb{E}[\boldsymbol{C}_2(0)] = 0$, we have $Const_2 = \frac{\Lambda_1}{(\Delta_1-\Delta_2)}$ and the solution for $i = 2$ reads

$$\mathbb{E}[\boldsymbol{C}_2(t)] = \Lambda_1\left[\frac{e^{-\Delta_1 t}}{(\Delta_2-\Delta_1)} + \frac{e^{-\Delta_2 t}}{(\Delta_1-\Delta_2)}\right].$$

Thus, in general for any $i = 2,\dots,N$,

$$\mathbb{E}[\boldsymbol{C}_i(t)] = \left(\prod_{l=1}^{i-1}\Lambda_l\right)\sum_{j=1}^{i} e^{-\Delta_j t}\prod_{\substack{m=1\\ m\neq j}}^{i}(\Delta_m-\Delta_j)^{-1}.$$

While we have considered here the case of different eigenvalues, the most general solution can be found in Ref[2], which models the radioactive decay of a chain of nuclides. For illustrative purposes, the case where all eigenvalues are the same is studied next.

Derivation of Eq. (6)

If $\Delta_i = \Delta_j = \Delta$ for all $i, j \in \{1,\dots,N\}$, system (A.1) for the irreversible model becomes

$$\begin{aligned}
\frac{d\,\mathbb{E}[\boldsymbol{C}_1(t)]}{dt} &= -\Delta\,\mathbb{E}[\boldsymbol{C}_1(t)], \\
\frac{d\,\mathbb{E}[\boldsymbol{C}_i(t)]}{dt} &= \Lambda_{i-1}\,\mathbb{E}[\boldsymbol{C}_{i-1}(t)] - \Delta\,\mathbb{E}[\boldsymbol{C}_i(t)], \\
\frac{d\,\mathbb{E}[\boldsymbol{C}_N(t)]}{dt} &= \Lambda_{N-1}\,\mathbb{E}[\boldsymbol{C}_{N-1}(t)] - \Delta\,\mathbb{E}[\boldsymbol{C}_N(t)].
\end{aligned} \tag{A.5}$$

Therefore, the solution of the second equation is given by

$$\begin{aligned}
\mathbb{E}[\boldsymbol{C}_2(t)] &= \left[\Lambda_1\int \mathbb{E}[\boldsymbol{C}_1(t)]\,e^{\Delta t}\,dt\;+Const_2\right]\,e^{-\Delta t} \\
&= \left[\Lambda_1\int 1\,dt\,e^{-\Delta t} + Const_2\,e^{-\Delta t}\right] \\
&= \left[\Lambda_1 t\,e^{-\Delta t} + Const_2\,e^{-\Delta t}\right] \\
&= \Lambda_1 t\,e^{-\Delta t}.
\end{aligned} \tag{A.6}$$

Here, we make use of $\mathbb{E}[\boldsymbol{C}_1(t)] = e^{-\Delta t}$ as in Eq. (A.2), and $Const_2 = 0$. Following a similar argument we can write

$$\mathbb{E}[\boldsymbol{C}_3(t)] = \Lambda_1\Lambda_2\,\frac{t^2}{2!}\,e^{-\Delta t},$$

in agreement with Eq. (6). The reader may notice that when substituting the exponential solution for the previous compartment, the product of exponentials in the integral on the right hand side is one. Thus, only the integration of a constant is required in each step; this generates the power t^{i-1}.

Derivation of Eq. (9)

Under the assumption $\Delta_i = \Delta_j = \Delta$ for all $i, j \in \{1, \ldots, N-1\}$, but with $\Delta_N = 0$, system (A.1) for the irreversible model becomes

$$\begin{aligned} \frac{d\,\mathbb{E}[\boldsymbol{C}_1(t)]}{dt} &= -\Delta\,\mathbb{E}[\boldsymbol{C}_1(t)], \\ \frac{d\,\mathbb{E}[\boldsymbol{C}_i(t)]}{dt} &= \Lambda_{i-1}\,\mathbb{E}[\boldsymbol{C}_{i-1}(t)] - \Delta\,\mathbb{E}[\boldsymbol{C}_i(t)], \\ \frac{d\,\mathbb{E}[\boldsymbol{C}_N(t)]}{dt} &= \Lambda_{N-1}\,\mathbb{E}[\boldsymbol{C}_{N-1}(t)]. \end{aligned} \tag{A.7}$$

The differential equation for $i = N$ is only a function of parameters and the variable relative to the previous compartment C_{N-1}; thus, the solution reads

$$\mathbb{E}[\boldsymbol{C}_N(t)] = \Lambda_{N-1} \int \mathbb{E}[\boldsymbol{C}_{N-1}(t)]\,dt + Const_N.$$

We recall that for $i = 1, \ldots, N-1$ the solution is given by,

$$\mathbb{E}[\boldsymbol{C}_i(t)] = \left(\prod_{l=1}^{i-1} \Lambda_l\right) \frac{t^{i-1}}{(i-1)!} e^{-\Delta t}, \quad t \geq 0. \tag{A.8}$$

Substituting for $i = N-1$ and integrating, we obtain

$$\begin{aligned} \mathbb{E}[\boldsymbol{C}_N(t)] &= \Lambda_{N-1} \int \left(\prod_{l=1}^{N-2} \Lambda_l\right) \frac{t^{N-2}}{(N-2)!} e^{-\Delta t} dt + Const_N \\ &= \Lambda_{N-1} \left[\frac{1}{-\Delta} \left(\prod_{l=1}^{N-2} \Lambda_l\right) \frac{t^{N-2}}{(N-2)!} e^{-\Delta t} + \frac{1}{\Delta} \left(\prod_{l=1}^{N-2} \Lambda_l\right) \int \frac{t^{N-3}}{(N-3)!} e^{\Delta t} dt \right] + Const_N \\ &= -\frac{\Lambda_{N-1}}{\Delta} \mathbb{E}[\boldsymbol{C}_{N-1}(t)] + \frac{\Lambda_{N-1}\Lambda_{N-2}}{\Delta} \left(\prod_{l=1}^{N-3} \Lambda_l\right) \int \frac{t^{N-3}}{(N-3)!} e^{\Delta t} dt + Const_N \\ &= -\frac{\Lambda_{N-1}}{\Delta} \mathbb{E}[\boldsymbol{C}_{N-1}(t)] + \frac{\Lambda_{N-1}\Lambda_{N-2}}{\Delta} \left(\prod_{l=1}^{N-3} \Lambda_l\right) \left[\frac{-1}{\Delta} \frac{t^{N-3}}{(N-3)!} e^{\Delta t} + \frac{1}{\Delta} \int \frac{t^{N-4}}{(N-4)!} e^{\Delta t} dt \right] + Const_N \\ &= -\frac{\Lambda_{N-1}}{\Delta} \mathbb{E}[\boldsymbol{C}_{N-1}(t)] - \frac{\Lambda_{N-1}\Lambda_{N-2}}{\Delta\Delta} \mathbb{E}[\boldsymbol{C}_{N-2}] + \frac{\Lambda_{N-1}\Lambda_{N-2}\Lambda_{N-3}}{\Delta} \left(\prod_{l=1}^{N-4} \Lambda_l\right) \int \frac{t^{N-4}}{(N-4)!} e^{\Delta t} dt + Const_N \\ &= -\frac{\Lambda_{N-1}}{\Delta} \mathbb{E}[\boldsymbol{C}_{N-1}(t)] - \frac{\Lambda_{N-1}\Lambda_{N-2}}{\Delta\Delta} \mathbb{E}[\boldsymbol{C}_{N-2}] - \cdots - \left(\prod_{l=1}^{N-1} \frac{\Lambda_l}{\Delta}\right) \mathbb{E}[\boldsymbol{C}_1(t)] + Const_N. \end{aligned}$$

Given the initial conditions $\mathbb{E}[\boldsymbol{C}_N(0)] = \mathbb{E}[\boldsymbol{C}_{N-1}(0)] = \cdots = \mathbb{E}[\boldsymbol{C}_2(0)] = 0$ and $\mathbb{E}[\boldsymbol{C}_1(0)] = 1$, we find $Const_N = \prod_{l=1}^{N-1} \frac{\Lambda_l}{\Delta_l}$. Hence,

$$\mathbb{E}[\boldsymbol{C}_N(t)] = \prod_{i=1}^{N-1} \frac{\Lambda_i}{\Delta_i} - \sum_{j=1}^{N-1} \mathbb{E}[\boldsymbol{C}_j(t)] \prod_{i=j}^{N-1} \frac{\Lambda_i}{\Delta_i},$$

in agreement with Eq. (9).

B The progeny of a single progenitor cell: derivation of Eq. (16)

By the definition of the probability generating function, $\Psi_i(z) = \mathbb{E}(z^{S_i})$. Moreover, we let $E_j \in \{$*death, differentiation, self-renewal, asymmetric division, symmetric division*$\}$. We make use of Eq. (15), in particular

$$\begin{aligned} \Psi_i(z) &= \sum_{E_j} \mathbb{E}(z^{S_i} \,|\, event\, E_j) \mathbb{P}(E_j) \\ &= \mathbb{E}(z^{S_i} \,|\, death) \mathbb{P}(death) + \mathbb{E}(z^{S_i} \,|\, differentiation)\ \mathbb{P}(differentiation) \\ &\quad + \mathbb{E}(z^{S_i} \,|\, self\text{-}renewal) \mathbb{P}(self\text{-}renewal) + \mathbb{E}(z^{S_i} \,|\, asymmetric\ division)\ \mathbb{P}(asymmetric\ division) \\ &\quad + \mathbb{E}(z^{S_i} \,|\, symmetric\ division)\ \mathbb{P}(symmetric\ division). \end{aligned}$$

Now we observe that $\mathbb{E}(z^{S_i} \mid death) = z(\Psi_i(z))^0$ as, in case of first event being death, the initial cell cannot move to the next compartment; $\mathbb{E}(z^{S_i} \mid differentiation) = z(\Psi_{i+1}(z))$ as a single cell moves from compartment C_i to the next compartment C_{i+1}; $\mathbb{E}(z^{S_i} \mid self\text{-}renewal) = z(\Psi_i(z))^2$ as a cell in compartment C_i divides and the two daughter cells stay in the same compartment as the mother; $\mathbb{E}(z^{S_i} \mid asymmetric\ division) = z\Psi_i(z)\Psi_{i+1}(z)$ as when one cell divides by asymmetric division, one of the two daughter cells moves to the next compartment and one stays in the same compartment as the mother; and $\mathbb{E}(z^{S_i} \mid symmetric\ division) = z(\Psi_{i+1}(z))^2$ as when a cell in compartment C_i divides by symmetric division, the two daughter cells move to the following compartment C_{i+1}. Thus, we have

$$\begin{aligned}\Psi_i(z) = z(\Psi_i(z))^0\,\mathbb{P}(death) + z\Psi_{i+1}(z)\,\mathbb{P}(differentiation) + z(\Psi_i(z))^2\,\mathbb{P}(self\text{-}renewal)\\ + z\Psi_i(z)\Psi_{i+1}(z)\,\mathbb{P}(asymmetric\ division) + z(\Psi_{i+1}(z))^2\,\mathbb{P}(symmetric\ division).\end{aligned}$$

Making use of events rates and their probabilities, we get Eq. (16).

C Lifeline analysis

Derivation of τ_i (lifespan)

We consider the average lifespan of a tracked cell starting in compartment C_i; by making use of first-step arguments we obtain the following linear system of equations,

$$\begin{aligned}(\mu_1+\nu_1)\tau_1 &= \nu_1\tau_2+1,\\ (\mu_i+\nu_i+\xi_i)\tau_i &= \nu_i\tau_{i+1}+\xi_i\tau_{i-1}+1, \quad i\in\{1,\dots,N-1\},\\ (\mu_N+\xi_N)\tau_N &= \xi_N\tau_{N-1}+1.\end{aligned}$$

This can be written in a matrix form as follows

$$\begin{pmatrix} \bar{\Delta}_1^{-1} & -\nu_1 & 0 & 0 & \cdots & 0\\ -\xi_2 & \bar{\Delta}_2^{-1} & -\nu_2 & 0 & \cdots & 0\\ 0 & -\xi_3 & \bar{\Delta}_3^{-1} & -\nu_3 & \cdots & 0\\ \vdots & \ddots & \ddots & \ddots & \ddots & \vdots\\ 0 & \cdots & 0 & -\xi_{N-1} & \bar{\Delta}_{N-1}^{-1} & -\nu_{N-1}\\ 0 & \cdots & 0 & 0 & -\xi_N & \bar{\Delta}_N^{-1} \end{pmatrix} \begin{pmatrix}\tau_1\\ \tau_2\\ \vdots\\ \tau_{n-1}\\ \tau_N\end{pmatrix} = \begin{pmatrix}1\\ 1\\ \vdots\\ 1\\ 1\end{pmatrix}, \tag{C.1}$$

where

$$\begin{aligned}\bar{\Delta}_1 &= \mu_1+\nu_1,\\ \bar{\Delta}_i &= \mu_i+\nu_i+\xi_i, \quad i\in\{2,\dots,N-1\},\\ \bar{\Delta}_N &= \mu_N+\xi_N.\end{aligned}$$

By application of the Thomas algorithm[3,4], we can find the coefficients $\bar{\gamma}_i$ and $\bar{\rho}_i$; in particular, we obtain the following recursive system of linear equations

$$\begin{aligned}\tau_N &= \bar{\rho}_N,\\ \tau_i &= \bar{\rho}_i - \bar{\gamma}_i\tau_{i+1}, \quad i\in\{1,\dots,N-1\}.\end{aligned}$$

This can be solved via backward substitution, which gives the general solution

$$\tau_i = \sum_{k=i}^{N}(-1)^{k-i}\bar{\rho}_k\left(\prod_{p=i}^{k-1}\bar{\gamma}_p\right),$$

with $\bar{\gamma}_1$ and $\bar{\rho}_1$ defined in Section 2.3.1.

Derivation of η_i: number of divisions on the lifeline

We now consider the number of division events along the lifespan of the tracked cell, starting in compartment C_i. Using first-step arguments, we obtain the following system of linear equations for its average value $\eta_i = \mathbb{E}[D_i]$,

$$\begin{aligned}(\lambda_1+\mu_1+\nu_1)\eta_1 &= \lambda_1(\eta_1+1)+\nu_1\eta_2,\\ (\lambda_i+\mu_i+\nu_i+\xi_i)\eta_i &= \lambda_i(\eta_i+1)+\nu_i\eta_{i+1}+\xi_i\eta_{i-1}, \quad i\in\{1,\dots,N-1\},\\ (\lambda_N+\mu_N+\xi_N)\eta_N &= \lambda_N(\eta_N+1)+\xi_N\eta_{N-1}.\end{aligned}$$

This system can be written in matrix form as

$$\begin{pmatrix} \bar{\Delta}_1^{-1} & -\nu_1 & 0 & 0 & \cdots & 0 \\ -\xi_2 & \bar{\Delta}_2^{-1} & -\nu_2 & 0 & \cdots & 0 \\ 0 & -\xi_3 & \bar{\Delta}_3^{-1} & -\nu_3 & \cdots & 0 \\ \vdots & \ddots & \ddots & \ddots & \ddots & \vdots \\ 0 & \cdots & 0 & -\xi_{N-1} & \bar{\Delta}_{N-1}^{-1} & -\nu_{N-1} \\ 0 & \cdots & 0 & 0 & -\xi_N & \bar{\Delta}_N^{-1} \end{pmatrix} \begin{pmatrix} \eta_1 \\ \eta_2 \\ \vdots \\ \eta_{N-1} \\ \eta_N \end{pmatrix} = \begin{pmatrix} 0 \\ 0 \\ \vdots \\ 0 \\ \mu_N \end{pmatrix}. \tag{C.2}$$

We can make use of the Thomas algorithm[3,4] to solve this tri-diagonal system of equations, and find the following recursive form

$$\begin{aligned} \eta_N &= \tilde{\rho}_N, \\ \eta_i &= \tilde{\rho}_i - \bar{\gamma}_i \eta_{i+1}. \end{aligned}$$

Solving this system via backward substitution leads to the general solution

$$\eta_i = \sum_{k=i}^{N} (-1)^{k-i} \tilde{\rho}_k \left(\prod_{p=i}^{k-1} \bar{\gamma}_p \right), \tag{C.3}$$

with $\tilde{\rho}_k$ and $\bar{\gamma}_p$ defined as in Section 2.3.2.

Derivation of $\omega_i(n) \equiv \mathbb{P}(D_i = n)$

When looking at $\omega_i(n)$, defined as the probability that a single cell starting in compartment C_i divides exactly n times before it dies or leaves the system, for any non-negative integer n, we can define the associated linear system of equations. In particular, for illustrative purposes, let us consider $n = 2$, so that $\omega_i(2) \equiv \mathbb{P}(D_i = 2)$ is the probability that a single cell starting in compartment C_i divides exactly twice before it dies or leaves the system. In this case, the system written in matrix form reads as,

$$\begin{pmatrix} \hat{\Delta}_1^{-1} & -\nu_1 & 0 & 0 & \cdots & 0 \\ -\xi_2 & \hat{\Delta}_2^{-1} & -\nu_2 & 0 & \cdots & 0 \\ 0 & -\xi_3 & \hat{\Delta}_3^{-1} & -\nu_3 & \cdots & 0 \\ \vdots & \ddots & \ddots & \ddots & \ddots & \vdots \\ 0 & \cdots & 0 & -\xi_{N-1} & \hat{\Delta}_{N-1}^{-1} & -\nu_{N-1} \\ 0 & \cdots & 0 & 0 & -\xi_N & \hat{\Delta}_N^{-1} \end{pmatrix} \begin{pmatrix} \omega_1(2) \\ \omega_2(2) \\ \vdots \\ \omega_{N-1}(2) \\ \omega_N(2) \end{pmatrix} = \begin{pmatrix} \lambda_1 \omega_1(1) \\ \lambda_2 \omega_2(1) \\ \vdots \\ \lambda_{N-1} \omega_{N-1}(1) \\ \lambda_N \omega_N(1) \end{pmatrix}. \tag{C.4}$$

This leads to the following recursive system

$$\begin{aligned} \omega_N(2) &= \hat{\rho}_N(2), \\ \omega_i(2) &= \hat{\rho}_i(2) - \hat{\gamma}_i \omega_{i+1}(2), \end{aligned}$$

where $\hat{\Delta}_i = \mu_i + \lambda_i + \xi_i + \nu_i$, $\hat{\rho}_1(2) = \lambda_1 \omega_1(1) \hat{\Delta}_1^{-1}$, $\hat{\gamma}_1 = -\nu_1 \hat{\Delta}_1^{-1}$, and

$$\hat{\rho}_i(2) = \frac{\lambda_i \omega_i(1) + \xi_i \hat{\rho}_{i-1}(2)}{\hat{\Delta}_i + \xi_i \hat{\gamma}_{i-1}}, \quad \hat{\gamma}_i = \frac{-\nu_i}{\hat{\Delta}_i + \xi_i \hat{\gamma}_{i-1}}.$$

One can solve the system via backward substitutions which leads to the following solution for the case $n = 2$,

$$\omega_i(2) = \sum_{k=i}^{N} (-1)^{k-i} \hat{\rho}_k(2) \left(\prod_{j=i}^{k-1} \hat{\gamma}_j \right), \quad i \in \{1, \ldots, N\}.$$

Derivation of $\beta_i(j)$

We now consider the probability of a tracked cell starting in compartment C_i to die in a given compartment C_j. In particular, and for illustrative purposes, let us consider $\beta_i(1)$, the probability of a tracked cell from compartment C_i dying in compartment C_1. Using a first-step argument, we can write the following linear system of equations,

$$\begin{aligned} (\mu_1 + \nu_1)\beta_1(1) &= \nu_1 \beta_2(1) + \mu_1, \\ (\mu_i + \nu_i + \xi_i)\beta_i(1) &= \nu_i \beta_{i+1}(1) + \xi_i \beta_{i-1}(1), \\ (\mu_N + \xi_N)\beta_N(1) &= \xi_N \beta_{N-1}(1), \end{aligned}$$

which can be written in matrix form as,

$$
\begin{pmatrix}
\bar{\Delta}_1^{-1} & -\nu_1 & 0 & 0 & \cdots & 0 \\
-\xi_2 & \bar{\Delta}_2^{-1} & -\nu_2 & 0 & \cdots & 0 \\
0 & -\xi_3 & \bar{\Delta}_3^{-1} & -\nu_3 & \cdots & 0 \\
\vdots & \ddots & \ddots & \ddots & \ddots & \vdots \\
0 & \cdots & 0 & -\xi_{N-1} & \bar{\Delta}_{N-1}^{-1} & -\nu_{N-1} \\
0 & \cdots & 0 & 0 & -\xi_N & \bar{\Delta}_N^{-1}
\end{pmatrix}
\begin{pmatrix}
\beta_1(1) \\ \beta_2(1) \\ \vdots \\ \beta_{N-1}(1) \\ \beta_N(1)
\end{pmatrix}
=
\begin{pmatrix}
\mu_1 \\ 0 \\ \vdots \\ 0 \\ 0
\end{pmatrix}. \tag{C.5}
$$

Making use of the Thomas algorithm[3,4], one can find the coefficients $\bar{\rho}_i$ and $\bar{\gamma}_i$; thus, this system can be written in a recursive form as

$$
\begin{aligned}
\beta_N(1) &= \bar{\rho}_N, \\
\beta_i(1) &= \bar{\rho}_i - \bar{\gamma}_i . \beta_{i+1}(1)
\end{aligned}
$$

Applying backward substitution we get the following relation

$$
\beta_i(1) = \sum_{k=i}^{N} (-1)^{k-i} \bar{\rho}_k(1) \left(\prod_{p=i}^{k-1} \bar{\gamma}_p \right) \quad i \in \{1, \ldots, N\}.
$$

Analogous arguments can be applied to compute the probability of death of a chosen cell in any compartment C_j, *i.e.*, $\beta_i(j)$, as shown in Section 2.3.3.

References

1. Zill, D. A first course in differential equations.(10thedn). *Ricar d Stratton, Los Angeles, USA* (2013).
2. Dreher, R. Modified Bateman solution for identical eigenvalues. *Annals Nucl. Energy* **53**, 427–438, DOI: https://doi.org/10.1016/j.anucene.2012.06.019 (2013).
3. Conte, S. & De Boor, C. *Elementary Numerical Analysis: An Algorithmic Approach* (McGraw-Hill, USA, 1972).
4. Thomas, L. H. Elliptic problems in linear difference equations over a network. *Watson Sci. Comput. Lab. Rept., Columbia Univ. New York* **1**, 71 (1949).
